# Evaluation of the diagnostic value of 64 simultaneously measured autoantibodies for early detection of gastric cancer

**DOI:** 10.1038/srep25467

**Published:** 2016-05-03

**Authors:** Simone Werner, Hongda Chen, Julia Butt, Angelika Michel, Phillip Knebel, Bernd Holleczek, Inka Zörnig, Stefan B. Eichmüller, Dirk Jäger, Michael Pawlita, Tim Waterboer, Hermann Brenner

**Affiliations:** 1 Division of Clinical Epidemiology and Aging Research, DKFZ, Heidelberg, Germany; 2 Division of Molecular Diagnostics of Oncogenic Infections, DKFZ, Heidelberg, Germany; 3 Department of General, Visceral and Transplantation Surgery, University of Heidelberg, Heidelberg, Germany; 4 Saarland Cancer Registry, Saarbrücken, Germany; 5 Department of Medical Oncology, National Center for Tumor Diseases (NCT) and Heidelberg University Hospital, Heidelberg, Germany; 6 GMP & T cell Therapy Unit, German Cancer Research Center (DKFZ), Heidelberg, Germany; 7 Division of Preventive Oncology, German Cancer Research Center (DKFZ) and National Center for Tumor Diseases (NCT), Heidelberg, Germany; 8 German Cancer Consortium (DKTK), German Cancer Research Center (DKFZ), Heidelberg, Germany

## Abstract

Autoantibodies against tumor-associated antigens (TAAs) have been suggested as biomarkers for early detection of gastric cancer. However, studies that systematically assess the diagnostic performance of a large number of autoantibodies are rare. Here, we used bead-based multiplex serology to simultaneously measure autoantibody responses against 64 candidate TAAs in serum samples from 329 gastric cancer patients, 321 healthy controls and 124 participants with other diseases of the upper digestive tract. At 98% specificity, sensitivities for the 64 tested autoantibodies ranged from 0–12% in the training set and a combination of autoantibodies against five TAAs (MAGEA4 + CTAG1 + TP53 + ERBB2_C + SDCCAG8) was able to detect 32% of the gastric cancer patients at a specificity of 87% in the validation set. Sensitivities for early and late stage gastric cancers were similar, while chronic atrophic gastritis, a precursor lesion of gastric cancer, was not detectable. However, the 5-marker combination also detected 26% of the esophageal cancer patients. In conclusion, the tested autoantibodies and combinations alone did not reach sufficient sensitivity for gastric cancer screening. Nevertheless, some autoantibodies, such as anti-MAGEA4, anti-CTAG1 or anti-TP53 and their combinations could possibly contribute to the development of cancer early detection tests (not necessarily restricted to gastric cancer) when being combined with other markers.

Despite decreasing incidence worldwide, almost one million people were estimated to have been diagnosed with gastric cancer in 2012, and most patients diagnosed with gastric cancer die from this disease^1^. Eradication of *Helicobacter pylori* infection, the most important risk factor of non-cardia gastric cancer, has been shown to reduce gastric cancer incidence and mortality in several randomized clinical trials^2^,^3^. Another approach to lower mortality would be early cancer detection. Screening by upper endoscopy has been implemented for individuals at high risk of gastric cancer^4^. A sensitive blood test would be an attractive alternative for both average-risk individuals from high-incidence countries and high risk groups from low-incidence countries^5^.

Autoantibodies against tumor-associated antigens (TAAs) have been found in serum of patients with various types of cancer and could serve as biomarkers for early detection of gastric cancer as well^6^,^7^,^8^,^9^,^10^. In a recent systematic literature review we have evaluated the potential of known autoantibody markers for this purpose^11^. However, direct comparison of different markers and marker combinations was difficult because of differences in the study populations and autoantibody detection methods. Furthermore, many promising autoantibody markers have not been independently validated so far.

In order to overcome these limitations, we designed a study including autoantibody measurements in over 800 serum samples from healthy controls, gastric cancer patients and persons with other diseases of the upper digestive tract by bead-based multiplex serology. With this method it is possible to measure up to 100 antibodies simultaneously, with performance characteristics comparable or even better than standard serological techniques like ELISA^12^.

The aim of our study was to identify autoantibody combinations that are able to detect a substantial proportion of gastric cancer patients at a reasonably high specificity.

## Methods

### Study design and study population

**Figure 1:**
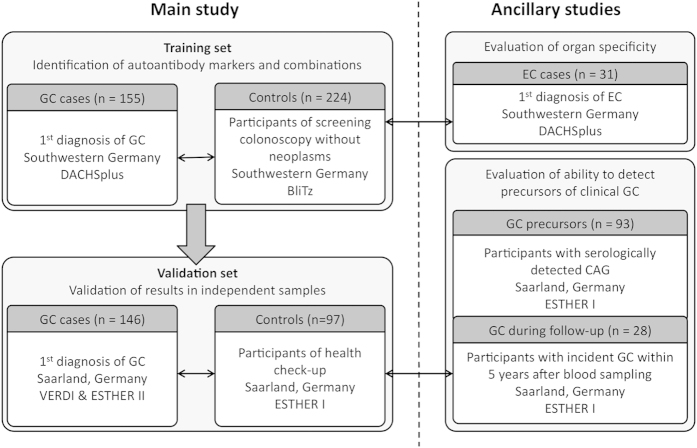
Study design and study population. Abbreviations: CAG = chronic atrophic gastritis, EC = esophageal cancer, GC = gastric cancer, n = number of participants with valid measurement results.

Our study followed a two-step approach with marker selection for gastric cancer detection in a training set (step 1) and subsequent evaluation of diagnostic performance of selected autoantibody markers and marker combinations in independent validation samples (step 2). In addition, we performed analyses with patients with other diseases of the upper digestive tract and participants with a diagnosis of gastric cancer during follow-up to assess disease specificity and the ability of autoantibody markers to detect precursors of clinical gastric cancer. An overview of the study design is shown in Fig.1, and detailed information on the studies from which the study populations were sampled is shown in the supplementary methods and Supplementary Table S1.

Gastric cancer cases included in the training set were recruited in southwestern Germany in the context of the DACHSplus study, a satellite substudy to the case-control study DACHS^13^,^14^,^15^. In addition to colorectal cancer (CRC) patients recruited for the DACHS study, patients with a primary diagnosis of other gastrointestinal cancers were enrolled in DACHSplus. Controls in the training set were selected from the BliTz study, an ongoing prospective CRC study among participants of screening colonoscopy, conducted in cooperation with more than 20 gastroenterology practices in the same geographic region as DACHS^16^,^17^,^18^.

Gastric cancer cases included in the validation set were recruited in the context of the ESTHER II study and the VERDI study^19^,^20^. These are two large unselected cancer patient cohort studies conducted in the entire state of Saarland, also located in the southwest of Germany. Controls of the validation set were recruited by their general practitioners in the ESTHER I study, a prospective cohort study among participants of general health check-up^21^.

For the main study all available gastric cancer patients from DACHSplus, ESTHER II and VERDI (n = 316) were included in the autoantibody measurements. The controls (n = 335) were randomly drawn from BliTz and ESTHER I after exclusion criteria were applied (see Supplementary Table 1 for eligibility criteria). For the additional analyses, we furthermore included esophageal cancer patients from DACHSplus (n = 35), chronic atrophic gastritis patients from ESTHER I (n = 100) as well as ESTHER I participants with a diagnosis of gastric cancer during the follow-up period (2002–2011, n = 29).

Because sex differences between subjects with and without gastric cancer (higher proportion of males in the former) would be expected in any screening population^1^ and as we intended to derive diagnostic performance estimates which are representative for the screening situation, we did not match cases and controls. However, we carried out a sensitivity analysis in a matched subset of the validation set as described in Supplementary Method 3. This way, the ability of autoantibody marker combinations to discriminate cases and controls independent of differences in the age and sex distributions could be assessed.

All studies were approved by the ethics committees of the University of Heidelberg and of the respective state medical boards. From each participant written informed consent was obtained. Methods were carried out in accordance with approved guidelines (e.g. good epidemiological practice, good laboratory practice).

### Sample collection and handling

For BliTz participants, serum samples were taken by gastroenterologists before screening colonoscopy and for ESTHER I participants, serum samples were taken by general practitioners during a general health examination. For 14 participants of ESTHER I who developed gastric cancer during the follow-up period, also 5 year follow-up blood samples were available. Blood samples from DACHSplus participants were taken in hospitals before surgery (but after neoadjuvant therapy for some patients). Blood samples from gastric cancer patients from VERDI and ESTHER II were taken in hospitals or at patients’ homes at various time points (before surgery: 25 patients, 1–14 days after surgery: 66 patients, 15–90 days after surgery: 36 patients, >90 days after surgery: 11 patients, no surgery or time of surgery unknown: 15 patients.) All obtained serum samples were stored at −80 °C until the autoantibody measurements.

### Bead-based multiplex serology measurements

We selected 64 candidate TAAs encoded by 59 genes based on previous autoantibody measurements in melanoma, ovarian cancer and pancreatic cancer patients^22^,^23^ and two systematic reviews^11^,^24^. Among them there were many cancer-germline antigens (e.g. CTAG1, CTAG2, DDX53, MAGEA1, MAGEA3, MAGEA4) and proteins from pathways known to be dysregulated in cancers (e.g. TP53, ERBB2, EGFR). Details on the 64 candidate TAAs are provided in Supplementary Table S2.

Autoantibodies against the selected TAAs were measured by multiplex serology, a fluorescent bead-based glutathione S-transferase (GST) capture immunosorbent assay, as described previously^12^,^23^. In short, TAAs were bacterially expressed as GST-X-tag fusion proteins^25^, loaded and affinity-purified on glutathione-casein-coupled spectrally distinct fluorescence-labeled polystyrene beads (SeroMap, Luminex Corp., Austin, Tx, USA). A mix of differently loaded bead sets provides an antigen suspension array that is presented to sera. A Luminex analyzer (Luminex Corp., Austin, Tx, USA) distinguishes the bead set by its internal bead-color and quantifies the amount of bound serum antibody detected by a secondary goat anti-human IgA, IgM, IgG antibody (Dianova, Hamburg, Germany) and the reporter conjugate streptavidin-R-phycoerythrin. The antibody reactivity is given as median fluorescence intensity (MFI) of at least 100 beads per set measured. Final antigen-specific MFI values were generated by subtraction of GST-tag and individual bead background values. Serum samples with a very high background fluorescence signal (reactivity for GST-tag >300 MFI) were excluded from further analyses.

Measurements of autoantibodies were performed in a 1:1000 dilution in the Division of Molecular Diagnostics of Oncogenic Infections, DKFZ, Heidelberg, Germany. In the first round of the antibody measurements 62 of the 64 autoantibodies were measured simultaneously. Anti-TP53 and anti-CDKN2A were measured together with antibodies against bacterial and viral antigens that are not subject of this analysis in the second round of the antibody measurements. For both measurements, the laboratory staff was blinded for the case-control status.

Antigen-loading of the beads was controlled via detection of the C-terminal tag and identity of the antigen loaded on the beads was verified by identifying the encoding plasmids via PCR and sequencing. Variation between different assay-plates was controlled by three control sera on each plate as replicates. Of these replicates, coefficients of variation (CV, = ratio of the standard deviation to the mean) were calculated for each antigen with a mean reactivity above 30 MFI. The median (range) CVs for all antigens combined in all three control sera were 16% (10–24%), 14% (11–21%), and 18% (11–25%), respectively.

### Statistical analysis

For characterization of the study population we used standard descriptive statistics and hypothesis tests (Fisher’s exact test, Wilcoxon rank-sum test). The strategy for selection of autoantibodies and their cutoffs for prediction of presence of gastric neoplasms was tailored to the particular character of single autoantibody markers, which typically have rather low sensitivity at very high specificity^11^,^24^.

Individual cutoffs for each autoantibody were calculated based on the MFI values of the controls from the training set: Blood samples with MFI values exceeding the 98^th^ percentile of the BliTz controls were considered as seropositive. To reduce noise from very weak fluorescence signals, cutoffs below 50 MFI were set to 50 MFI. Frequencies of autoantibodies against each antigen with 95% Wilson score confidence intervals (CIs)^26^ were determined for the different groups of study participants from the training and the validation set. As sensitivity analyses training and test set were swapped and cutoffs, sensitivities and specificities were calculated as aforementioned.

To evaluate the diagnostic performance of autoantibody combinations we generated all possible 2-, 3-, 4- and 5-marker combinations. A multi-marker test was considered as positive if the MFI value for at least one autoantibody of the combination was higher than the autoantibody-specific cutoff. All multi-marker combinations were ranked by Youden’s index (J = sensitivity + specificity −1) for the training set and for the marker combinations with the highest Youden’s indices, sensitivities and specificities with Wilson score intervals were evaluated in the validation set and in the additional samples (CAG, esophageal cancer, participants with diagnosis of gastric cancer during the follow-up period). Subgroup specific analyses were performed for early versus late stage cancers, cardia versus non-cardia gastric adenocarcinomas, men versus women, persons aged under 65 versus persons aged 65 and above and untreated patients versus patients who received radiotherapy, chemotherapy or surgery before blood withdrawal. To test for differences in the ability of the best 5-marker combination to correctly classify cases as cases and controls as controls between different subgroups Fisher’s exact test was used.

All analyses were performed with R (version 3.1.0)^27^. All statistical tests were two-sided and p-values below 0.05 were considered statistically significant.

## Results

### Study population characteristics

To study autoantibody responses against 64 TAAs we selected 829 serum samples from 815 individuals (gastric cancer patients, controls, esophageal cancer patients and CAG patients) and performed autoantibody measurements by bead-based multiplex serology. For 785 samples (95%) valid measurement results were obtained, while 44 samples had to be excluded due to insufficient amount of serum (n = 37) or high background fluorescence (n = 7). Participants with and without valid measurement results did not differ in respect of age, sex, stage, case-/control-status or study (each p-value > 0.05).

An overview of the study design and final numbers and characteristics of the different groups of participants are provided in Fig. 1 and Table 1. The mean age at recruitment was similar across all groups of participants and ranged from 61 years (controls ESTHER I study) to 66 years (gastric cancer patients ESTHER I study). With 66–68% and 81% the percentages of male subjects were significantly higher among gastric cancer and esophageal cancer cases than among controls (45–51%, p < 0.0001 and p = 0.0003, respectively). They were also higher than among CAG patients (47%, p = 0.0006 and p = 0.0015, respectively). For 23 gastric cancer patients from the training set, 27 gastric cancer patients from the validation set and all participants from ESTHER I with diagnosis of gastric cancer during the follow-up period, the UICC stage was unknown. About half of the remaining gastric cancer patients of the training set were diagnosed at an early stage (UICC 0-II), while in the validation set there were slightly more late-stage (UICC III-IV) gastric cancer patients. Due to recent changes in the gastric cancer treatment guidelines^28^, gastric cancer patients of the training set, who were more recently recruited in the context of the DACHSplus study, more often received neoadjuvant therapy than gastric cancer patients of the validation set (ESTHER II and VERDI study).

### Diagnostic performance of single autoantibody markers for gastric cancer detection

For each measured autoantibody an individual cutoff for seropositivity was calculated based on the MFI values of the controls from the training set (cutoff = 98^th^ percentile of controls, minimum 50 MFI). The cutoffs ranged from 50 MFI to 3633 MFI (see Supplementary Table S3). The highest cutoffs were observed for SPANXA, HIST1H2B and MPHOSPH6 indicating numerous autoantibody responses in healthy controls against those antigens. At cutoffs yielding at least 98% specificity, sensitivities for gastric cancer detection ranged from 0% to 12% in the training set and autoantibody responses were found in both early and late stage cancers. Antibody frequencies of at least five percent were seen for the antigens MAGEA4, CTAG1, CTAG2, DDX53, TP53, MAGEA3, SDCCAG8, KLK3_iso2, ERBB2_N, ERBB2_C, IGF2BP1, GRINA and UBQLN1 (see Table 2). In the validation set autoantibodies against MAGEA3, MAGEA4, CTAG1 and TP53 performed best with Youden’s indexes of 0.10 (anti-MAGEA3), 0.09 (anti-MAGEA4) and 0.07 (anti-CTAG1, anti-TP53).

**Figure 2:**
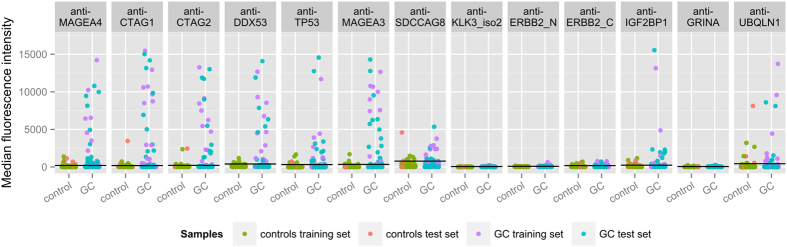
Median fluorescence intensities in gastric cancer cases and controls for the top 13 autoantibodies (based on sensitivity at 98% specificity in the training set).

**Figure 3:**
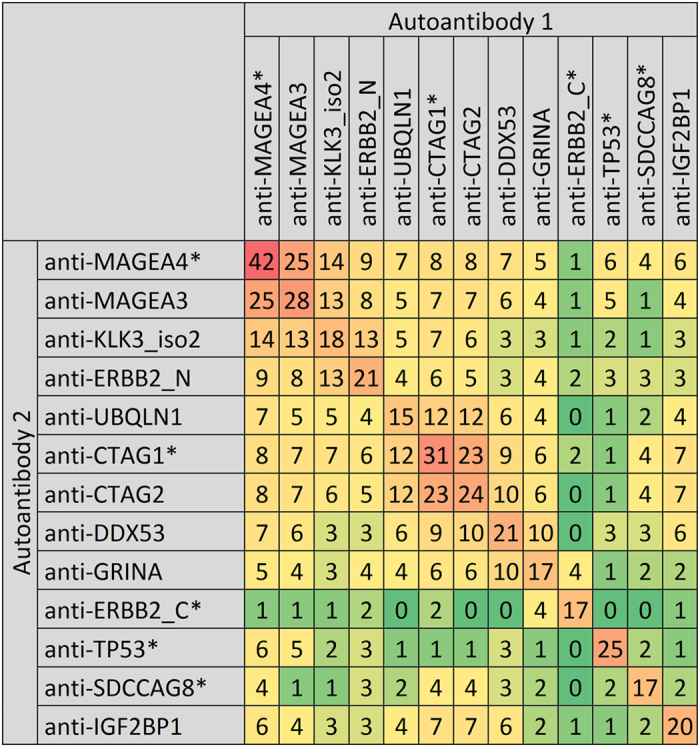
Absolute number of serum samples from gastric cancer patients (DACHSplus, ESTHER I, ESTHER II, VERDI) that were tested positive for 2 of the top 13 autoantibodies. The abundance of multiple autoantibodies is common among the tested gastric cancer cases.*Autoantibodies selected for 5-marker panel.

For some autoantibodies, antibody reactivities ranged from rather weak to very strong reactivities as measured e.g. for anti-CTAG1 with mean reactivity of 5108 MFI in seropositive gastric cancer cases, which is 28.3× higher than the cutoff (see Fig. 2). Moreover, many cancer patients developed autoantibodies directed against several of the TAAs (Fig. 3). Especially for antigens with high structural similarity (e.g. CTAG1 and CTAG2 or MAGEA3 and MAGEA4), a high correlation in seroreactivity was observed.

After swapping of the training and test set 4 of the top 5 autoantibodies and 9 of the top 13 autoantibodies from the old training set were also among the top 5 and top 13 autoantibodies in the new training set, respectively (see Supplementary Table S4).

### Diagnostic performance of marker combinations for gastric cancer detection

To evaluate the diagnostic performance of autoantibody combinations, we generated all possible 2-, 3-, 4- and 5-marker combinations and ranked them by the Youden’s index (J) for the training set. According to this criterion, the best 2-, 3-, 4- and 5- marker combinations were found for autoantibodies against MAGEA4 + CTAG1 (J = 0.15, 19% sensitivity, 96% specificity), MAGEA4 + CTAG1 + TP53 (J = 0.18, 25% sensitivity, 93% specificity), MAGEA4 + CTAG1 + TP53 + ERBB2_C (J = 0.21, 30% sensitivity, 91% specificity) and MAGEA4 + CTAG1 + TP53 + ERBB2_C + SDCCAG8 (J = 0.23, 34% sensitivity, 89% specificity). However, there are other 5-marker combinations that can detect gastric cancer patients with very similar sensitivities and specificities (see Table 3). Because specificity in the screening situation typically should not go below a limit of about 90%, we decided to not further increase the number of autoantibodies in a marker combination and to conduct the subsequent analyses only with the best performing five-marker panel.

All top 11 5-marker panels included the autoantibodies anti-MAGEA4, anti-CTAG1 and anti-TP53. Anti-ERBB2_C was included eight times, anti-SDCCAG8 was included four times and anti-DDX53 was included two times, while the other TAAs were included only once. After swapping of the training and test set new top 5-marker combinations were selected. However, all top 11 combinations also comprised anti-TP53 (see Supplementary Table S5).

### Diagnostic performance of a 5-marker panel in subgroups of gastric cancer patients and patients with other diseases of the upper digestive tract

The diagnostic performance of the best performing 5-marker panel was evaluated separately for gastric cancer patients from different studies and cancer stages (see Table 4). Early and late stage cancers were both detected with similar sensitivity (35% and 32% sensitivity at 87% specificity in the validation set) and no significant differences in diagnostic performance were found between gastric cancer patients from ESTHER II and VERDI (p = 0.59). Interestingly, some of the few gastric cancer patients from the cohort study ESTHER I presented autoantibodies against TAAs several years before gastric cancer diagnosis (for details see Supplementary Table S7). The 5-marker panel was also able to detect 26% of the esophageal cancer patients. However, patients with chronic atrophic gastritis, which is a precursor of gastritis cancer, were not detected by the 5-marker panel (see Table 4).

To evaluate the ability of the best performing 5-marker panel to discriminate gastric cancer cases and controls independent of differences in the age and sex distributions we performed a sensitivity analyses in a matched subset of the validation set (120 gastric cancer cases, 97 controls) using adjustment weights. With age- and sex adjusted sensitivities and specificities of 33% and 88% diagnostic performance characteristics after adjustment were very similar to the unadjusted diagnostic performance characteristics. In accordance with this result, no significant differences between men and women or persons under 65 years and persons aged 65 and above were found in the subgroup analyses (see Supplementary Table S6). Likewise, subgroup analyses in untreated patients versus patients that received radiotherapy, chemotherapy or surgery before blood withdrawal, cardia versus non-cardia gastric cancers, persons with different times of blood withdrawal in relation to surgery or different *H. pylori* infection status did not reveal significant differences between those groups.

## Discussion

We measured autoantibodies against 64 candidate TAAs by bead-based multiplex serology in serum samples from 329 gastric cancer patients, 321 healthy controls and 124 participants with other diseases of the upper digestive tract. Sensitivities for gastric cancer detection for single autoantibodies ranged from 0–12% at 98% specificity in the training set, and a combination of five autoantibodies was able to detect about a third of the gastric cancer patients at a specificity of 87% in the validation set. Early stage cancers were detected with similar sensitivities as late stage cancers and in some patients autoantibodies were even found several years before gastric cancer diagnosis. Sensitivities for the detection of CAG and esophageal cancer by the 5-marker panel were 12% and 26%, respectively.

We selected many autoantibodies for our measurements based on promising diagnostic performance for early detection of cancer in two previously performed systematic literature reviews^11^,^24^. However, in our measurements the sensitivities observed for these autoantibodies were often considerably lower than those reported originally. The most contrary finding to previously published results was observed in case of autoantibodies against MTDH, also known as AEG-1, which have been described to be present in none of the controls but 59% of the gastric cancer cases^29^, compared to 2–3% in both gastric cancer cases and controls here. Some of the observed differences in diagnostic performance might be attributable to differences in the methods used to quantify autoantibodies, others to shortcomings in the study design and data analyses in former studies.

It is a disappointing but common phenomenon that initially promising candidate cancer biomarkers do not pass validation studies^30^,^31^. Possible explanations are that observed differences in serum levels of a certain biomarker in initial studies are not caused by the cancer but by differences in the study populations (e.g. different age and sex distributions in cases and controls) or in the blood sampling and storage conditions (e.g. different blood sample processing time for cases and controls)^32^. If the diagnostic performance of marker combinations is evaluated on the same data that were used to select the markers, results will be overoptimistic due to overfitting^33^. A similar effect can occur if specificity is determined on the same controls that were used to calculate the cutoffs for seropositivity^34^,^35^.

We tried to avoid overfitting and overoptimistic performance estimates by using gastric cancer cases and controls from independent studies for marker selection and validation of the 5-marker panels. To test the validity of our results, we furthermore performed a sensitivity analysis in which we swapped the training and the test set. For single autoantibodies there was a large overlap between the top markers identified with the original sets and with the swapped sets. These autoantibodies were also frequently selected for the best 5-marker combinations generated from both sets which demonstrates the robustness of our marker selection approach. After swapping training and test sets, Youden’s indexes of the top 5-marker combinations were higher for the training set and mostly lower for the validation set. This is not surprising in consideration of the fact that sample sizes for the swapped training set were smaller than for the original training set and supports our decision to pick the BliTz and DACHSplus participants as training set in the main analyses.

To our knowledge, there are only few other studies that have evaluated autoantibody combinations for the early detection of gastric cancer so far. Recently, two articles from the same university in China have been published that describe studies that tested combinations of autoantibodies against Koc ( = IGF2BP3), p62 ( = IGF2BP2), Imp1 ( = IGF2BP1), Cyclin B1 ( = CCNB1), p16 ( = CDKN2A), Survivin ( = BIRC5), c-myc ( = MYC) and p53 ( = TP53) for the early detection of gastric cardia adenocarcinoma^36^ or multiple cancer types including gastric cancer^9^. With a 7-marker combination Zhou *et al.* reported identifying 64% of all cases with gastric cardia adenocarcinoma (specificity: 86%)^36^ and the 8-marker combination of Wang *et al.* was reported to yield 56% sensitivity for gastric cancer detection at 86% specificity^9^. Seven of these eight autoantibodies were also included in our measurements but only anti-TP53 and anti-IGFBP1 were selected in the top 11 5-marker combinations. However, the apparently better diagnostic performances of shared autoantibodies in the two articles have to be viewed with caution because of small sample sizes and the fact that cutoffs were chosen based on the same controls that subsequently were used to calculate specificity. Furthermore, Wang *et al.* did not provide study populations characteristics or a detailed description of blood sampling and storage conditions^9^ which limits judgement of comparability of cases and controls in regard to these factors. In another autoantibody panel for gastric cancer early detection small peptides instead of full length proteins were used as antigens. The reported cross-validated sensitivity for a signature of 45 autoantibodies was 44% at a specificity of 90%^37^.

In our measurements autoantibodies were found to be not restricted to gastric cancer patients with certain cancer stages or gastric cancer subtypes. Moreover, the autoantibody measurements in esophageal cancer patients showed that autoantibodies in our best 5-marker combination can also be present in another cancer of the gastrointestinal tract. A comparison with the improved version of EarlyCDT^®^-Lung, a commercial autoantibody test for lung cancer early detection^38^,^39^, reveals big overlap between our best performing autoantibodies and the autoantibodies of this test: four of the seven autoantibodies used in the EarlyCDT^®^-Lung test (anti-MAGEA4, anti-CTAG1 = anti-NY-ESO-1, anti-DDX53 = anti-CAGE and anti-TP53) were among the five autoantibodies with the highest sensitivities for gastric cancer; the other three autoantibodies (anti-SOX2, anti-GBU4-5 and anti-HuD) were not included in our multiplex serology measurements. Also the autoantibody measurements by Wang *et al.* support the idea that many commonly measured autoantibodies are present in different types of cancer^9^.

With this knowledge, it seems likely that some of the false positive results from the control group indeed are true positive results representing patients that suffer from an undiagnosed other cancer, for example prostate cancer, which is the most frequently present but often undiagnosed cancer in old men in developed countries^1^. In accordance with this hypothesis is the observation that observed specificities in old persons (for whom cancer prevalence is likely to be higher) tended to be a bit lower than in young persons.

With our 5-marker panel as well as with the EarlyCDT^®^-Lung test only the minority of the cancer patients are detected at specificities of around 90%^39^. So, the question arises why the majority of cancer patients are missed. Is it just an issue of suboptimal autoantibody selection in current panels? Or do not all cancers lead to detectable immune responses? In general, there are two types of tumor antigens: neo-antigens that represent mutated tumor proteins and non-mutated self-antigens that are derived from proteins that are not present or present at lower levels in normal cells, e.g. cancer-germline antigens^40^. However, as neo-antigens are unique for each tumor, all important antigens we measured autoantibodies against, belonged to the second group. Even if there were mutations present in a reasonable percentage of the gastric cancer patients, it couldn’t be guaranteed that those mutations would lead to the development of autoantibodies, because immune recognition is dependent on the ability of peptides that carry the mutation to be transported and bound to MHC receptors^40^. The possibilities of combining autoantibody markers are further limited by the fact that many cancer-germline antigens are closely related and patient sera often react with either all or none of these TAAs. For instance autoantibodies against CTAG1 and CTAG2 or against MAGEA3 and MAGEA4 are frequently found together and the combination of such a pair of autoantibodies would not result in a large gain of sensitivity, while for example the addition of anti-TP53 to an autoantibody against a cancer-germline antigen could increase sensitivity substantially.

In addition to searching for new algorithms and autoantibody markers that complement the currently known autoantibody markers, there might be a high potential in combining known autoantibody markers with other candidate biomarkers for gastric cancer. Those could be traditional tumor markers like CEA, CA19-9 and CA72-4^41^, markers related to chronic atrophic gastritis (e.g. *H. pylori* antibodies and pepsinogens)^42^, TAAs or other proteins^43^,^44^, microRNAs^45^,^46^ or glycosylation signatures^47^.

To our knowledge, this is the first study in which simultaneous measurements by bead-based multiplex-serology were performed to systematically assess the diagnostic value of autoantibodies for the early detection of gastric cancer. There are specific strengths and limitations that have to be considered. Strengths are the simultaneousness of measurements, which allows a direct comparison of autoantibody markers, the large sample size (329 gastric cancer patients and 321 controls with valid measurements in total) and the use of independent training and test sets for marker selection and validation. However, both cases of the training set and the validation set were recruited in clinics after gastric cancer diagnosis (and initial treatment for some patients) and controls of the training set came from a setting optimized for colorectal cancer screening studies rather than for gastric cancer screening studies. Although autoantibodies are known to be stable and enduring^48^ and we did not observe significant differences in the diagnostic performance of the 5-marker panel between subgroups of gastric cancer patients that did or did not receive neoadjuvant therapy before blood withdrawal, it cannot be ruled out that diagnostic or therapeutic interventions or lifestyle changes in response to the gastric cancer diagnosis have influenced autoantibody levels. Furthermore, the number of prediagnostic gastric cancer cases from the ESTHER I study, in which autoantibodies were detected several years before gastric cancer diagnosis, is too small to decide if these are true results or chance findings. Larger prospective studies would be necessary to answer this question.

Further topics that should be addressed in future studies are the biological role and the dynamics of autoantibodies in cancer. For example, we observed that strength of the autoantibody responses varied largely among the markers with highest sensitivities in the training set. Further studies should explore if strong and weak autoantibody responses have the same diagnostic values for early cancer detection and if strength of an autoantibody response varies in the course of the progression from a premalignant lesion to a symptomatic cancer.

In conclusion, we have conducted large scale autoantibody measurements in serum samples from gastric cancer cases and controls. The tested autoantibodies and combinations alone did not reach sufficient sensitivity for gastric cancer screening. However, with moderate sensitivities and very high specificities, some of the tested autoantibodies, e.g. anti-MAGEA4, anti-CTAG1, or anti-TP53 could be good candidates for combinations with other cancer biomarkers. As autoantibodies seem not to be specific for a certain cancer type, autoantibodies against TAAs might be particularly useful for the development of a potential minimally invasive “universal cancer test” which might serve for preliminary unspecific cancer screening to select people who would most likely benefit from (typically more costly and complex) screening for specific cancers.

## Additional Information

**How to cite this article**: Werner, S. *et al.* Evaluation of the diagnostic value of 64 simultaneously measured autoantibodies for early detection of gastric cancer. *Sci. Rep.* **6**, 25467; doi: 10.1038/srep25467 (2016).

## Supplementary Material

Supplementary Information

## Figures and Tables

**Table 1:** Study population characteristics.

Characteristic	Samples training set		Samples validation set		Additional samples		
	GC DACHSplus	Controls BliTz	GC VERDI, ESTHER II	Controls ESTHER I	GC ESTHER I	CAG ESTHER I	EC DACHSplus
n	155	224	146	97	28*	93	31
Age at recruitment, mean ± SD, years	63.43 ± 11.83	62.12 ± 6.81	63.65 ± 10.15**	61.03 ± 6.65	65.57 ± 6.12	64.17 ± 6.25	62.71 ± 8.67
Sex							
male, n (%)	106 (68%)	101 (45%)	95 (65%)	49 (51%)	19 (68%)	44 (47%)	25 (81%)
female, n (%)	49 (32%)	123 (55%)	49 (34%)	48 (49%)	9 (32%)	49 (53%)	6 (19%)
unknown, n (%)	0 (0%)	0 (0%)	2 (1%)	0 (0%)	0 (0%)	0 (0%)	0 (0%)
Stage							
UICC 0	0 (0%)	NA	1 (1%)	NA	0 (0%)	NA	0 (0%)
UICC I	35 (23%)	NA	23 (16%)	NA	0 (0%)	NA	13 (42%)
UICC II	29 (19%)	NA	27 (18%)	NA	0 (0%)	NA	9 (29%)
UICC III	43 (28%)	NA	26 (18%)	NA	0 (0%)	NA	6 (19%)
UICC IV	25 (16%)	NA	42 (29%)	NA	0 (0%)	NA	1 (3%)
unknown	23 (15%)	NA	27 (18%)	NA	28 (100%)	NA	2 (6%)
GC subtype							
AEG	64 (41%)	NA	28 (19%)	NA	0 (0%)	NA	NA
NCGA	54 (35%)	NA	92 (63%)	NA	0 (0%)	NA	NA
other/unknown	37 (24%)	NA	26 (18%)	NA	28 (100%)	NA	NA
*H. pylori* infection							
*H. pylori-*	56 (36%)	66 (29%)	31 (21%)	47 (48%)	5 (18%)	21 (23%)	0 (0%)
*H. pylori + ,* CagA−	34 (22%)	41 (18%)	11 (8%)	27 (28%)	3 (11%)	17 (18%)	0 (0%)
*H. pylori + ,* CagA+	58 (37%)	30 (13%)	36 (25%)	23 (24%)	19 (68%)	55 (59%)	1 (3%)
unknown	7 (5%)	87 (39%)	68 (47%)	0 (0%)	1 (4%)	0 (0%)	30 (97%)
Neoadj. Th.							
yes	89 (57%)	NA	11 (8%)	NA	0 (0%)	NA	22 (71%)
no	66 (43%)	NA	127 (87%)	NA	28 (100%)	NA	9 (29%)
unknown	0 (0%)	NA	8 (5%)	NA	0 (0%)	NA	0 (0%)

Abbreviations: AEG = adenocarcinoma of the esophagogastric junction, CAG = chronic atrophic gastritis, EC = esophageal cancer, GC = gastric cancer, n = number, NCGA = noncardia gastric adenocarcinoma, Neoadj. Th. = neoadjuvant chemo- or radio-therapy before blood withdrawal, SD = standard deviation; *27 with valid measurement of the baseline blood sample, 12 with valid measurement of the 5-year follow-up blood sample; **for 3 ESTHER II participants the age was unknown.

**Table 2:** Diagnostic performance of the top 13 autoantibody markers for detecting gastric cancer.

Antigen	Training set		Validation set		
	Specificity [95% CI] in %	sensitivity [95% CI] in %	Youden’s index*	specificity [95% CI] in %	sensitivity [95% CI] in %
MAGEA4**	98 [95–99]	12 [7–18]	0.09	95 [88–98]	14 [10–21]
CTAG1**	98 [95–99]	11 [7–17]	0.07	98 [93–99]	9 [5–15]
CTAG2	98 [95–99]	8 [4–13]	0.05	98 [93–99]	8 [4–13]
DDX53	98 [95–99]	8 [4–13]	0.05	99 [94–100]	6 [3–11]
TP53**	98 [95–99]	7 [4–12]	0.07	99 [94–100]	8 [4–13]
MAGEA3	98 [95–99]	7 [4–12]	0.10	100 [96–100]	10 [6–16]
SDCCAG8**	98 [95–99]	6 [3–11]	0.02	97 [91–99]	5 [2–10]
KLK3_iso2	98 [95–99]	6 [3–11]	0.03	98 [93–99]	5 [2–10]
ERBB2_N	98 [95–99]	5 [3–10]	0.02	95 [88–98]	7 [4–12]
ERBB2_C**	98 [95–99]	5 [3–10]	0.02	98 [93–99]	4 [2–9]
IGF2BP1	98 [95–99]	5 [3–10]	0.01	94 [87–97]	7 [4–12]
GRINA	98 [95–99]	5 [3–10]	0.03	98 [93–99]	5 [3–10]
UBQLN1	98 [95–99]	5 [3–10]	0.02	97 [91–99]	5 [2–10]

**Table 3:** Diagnostic performance of the top 11 5-marker combinations*.

No.	Autoantibodies	Training set			Validation set		
		Youden’s index	sensitivity [95% CI] in %	specificity [95% CI] in %	Youden’s index	sensitivity [95% CI] in %	specificity [95% CI] in %
**1**	anti-MAGEA4 + anti-CTAG1 + anti-TP53 + anti-ERBB2_C + anti-SDCCAG8	0.23	34 [27–41]	89 [85–93]	0.19	32 [25–40]	87 (78-92)
**2**	anti-MAGEA4 + anti-CTAG1 + anti-TP53 + anti-ERBB2_C + anti-ANXA4	0.22	32 [25–40]	90 [85–93]	0.18	32 [25–39]	87 (78-92)
**3**	anti-MAGEA4 + anti-CTAG1 + anti-TP53 + anti-SDCCAG8 + anti-GRINA	0.22	32 [25–39]	90 [86–93]	0.18	32 [25–39]	87 (78-92)
**4**	anti-MAGEA4 + anti-CTAG1 + anti-TP53 + anti-SDCCAG8 + anti-TPM3_iso3	0.22	32 [25–40]	89 [85–93]	0.17	30 [23–38]	87 (78-92)
**5**	anti-MAGEA4 + anti-CTAG1 + anti-TP53 + anti-ERBB2_C + anti-DDX53	0.22	32 [25–40]	89 [85–93]	0.21	32 [25–40]	89 (81-94)
**6**	anti-MAGEA4 + anti-CTAG1 + anti-TP53 + anti-ERBB2_C + anti-PSCA	0.21	30 [24–38]	91 [87–94]	0.21	31 [24–39]	90 (82-94)
**7**	anti-MAGEA4 + anti-CTAG1 + anti-TP53 + anti-ERBB2_C + anti-FOLH1_iso1	0.21	30 [24–38]	91 [87–94]	0.19	29 [23–37]	90 (82-94)
**8**	anti-MAGEA4 + anti-CTAG1 + anti-TP53 + anti-ERBB2_C + anti-FOLH1_iso7	0.21	30 [24–38]	91 [87–94]	0.19	29 [23–37]	90 (82-94)
**9**	anti-MAGEA4 + anti-CTAG1 + anti-TP53 + anti-ERBB2_C + anti-DCT	0.21	30 [24–38]	91 [87–94]	0.19	29 [23–37]	90 (82-94)
**10**	anti-MAGEA4 + anti-CTAG1 + anti-TP53 + anti-SDCCAG8 + anti-DDX53	0.21	32 [25–39]	90 [85–93]	0.19	32 [25–39]	88 (80-93)
**11**	anti-MAGEA4 + anti-CTAG1 + anti-TP53 + anti-ERBB2_C + anti-IGF2BP1	0.21	32 [25–39]	90 [85–93]	0.16	33 [26–41]	84 (75-90)

*The 11 5-marker combinations were selected based on a maximum Youden’s index (J = sensitivity + specificity-1) in the training set. Anti-TP53, anti-MAGEA4 and anti-CTAG1 are present in all 11 combinations.

**Table 4:** Diagnostic performance of autoantibodies against MAGEA4 + CTAG1 + TP53 + SDCCAG8 + ERBB2_C for the detection of gastric cancer, esophageal cancer and chronic atrophic gastritis.

Group	n all	n positive	n negative	Sensitivity[95% CI] in %	Specificity[95% CI] in %
Controls training set	224	24	200		89 [85–93]
GC training set					
all	155	52	103	34 [27–41]	
early stage	64	20	44	31 [21–43]	
late stage	68	25	43	37 [26–49]	
Controls validation set	97	13	84		87 [78–92]
GC validation set					
all	146	47	99	32 [25–40]	
early stage	51	18	33	35 [24–49]	
late stage	68	22	46	32 [22–44]	
ESTHER II study	81	28	53	35 [25–45]	
VERDI study	65	19	46	29 [20–41]	
GC during follow-up					
Baseline blood samples	27	5	22	19 [8–37]	
FU5 blood samples	12	4	8	33 [14–61]	
Esophageal cancer	31	8	23	26 [14–43]	
CAG	93	11	82	12 [7–20]	

Abbreviations: n = number, GC = gastric cancer, CAG = chronic atrophic gastritis, FU5 = 5-year follow-up.
