# Supplementary material for: Evaluation of the diagnostic value of 64 simultaneously measured autoantibodies for early detection of gastric cancer
Source: Sci Rep. 2016 May 3;6:25467. doi: 10.1038/srep25467 (PMC4853774; doi:10.1038/srep25467)
Supplement: Supplementary Information [file srep25467-s1.doc]

Evaluation of the diagnostic value of 64 simultaneously measured autoantibodies for early detection of gastric cancer:

Supplementary information

**Authors:** Simone Werner^1^, Hongda Chen^1^, Julia Butt^2^, Angelika Michel^2^, Phillip Knebel^3^, Bernd Holleczek^4^, Inka Zörnig^5^, Stefan B. Eichmüller^6^, Dirk Jäger^5^, Michael Pawlita^2^, Tim Waterboer^2^, Hermann Brenner*^1,7,8^

* Corresponding author

**Affiliations:** ^1^Division of Clinical Epidemiology and Aging Research, DKFZ, Heidelberg, Germany; ^2^Division of Molecular Diagnostics of Oncogenic Infections, DKFZ, Heidelberg, Germany; ^3^Department of General, Visceral and Transplantation Surgery, University of Heidelberg, Heidelberg, Germany; ^4^Saarland Cancer Registry, Saarbrücken, Germany; ^5^Department of Medical Oncology, National Center for Tumor Diseases (NCT) and Heidelberg University Hospital, Heidelberg, Germany; ^6^GMP & T cell Therapy Unit, German Cancer Research Center (DKFZ), Heidelberg, Germany; ^7^Division of Preventive Oncology, German Cancer Research Center (DKFZ) and National Center for Tumor Diseases (NCT), Heidelberg, Germany; ^8^German Cancer Consortium (DKTK), German Cancer Research Center (DKFZ), Heidelberg, Germany

**Address for correspondence:** Hermann Brenner, MD, MPH, Division of Clinical Epidemiology and Aging Research (C070), German Cancer Research Center, Im Neuenheimer Feld 581, D-69120 Heidelberg, Germany, Phone +49-6221-421300, fax +49-6221-421302, E-mail h.brenner@dkfz.de

**Contents**

1. Supplementary methods
   - Method S1: Data collection
   - Method S2: Classification of cancer and chronic atrophic gastritis
   - Method S3: Sensitivity analysis for the assessment of the ability of the best performing 5-marker panel to discriminate gastric cancer cases and controls independent of differences in the age and sex distributions
2. Supplementary tables

- Table S1. Overview studies.
- Table S2. Symbols, official and alternative names of tumor-associated antigens used for autoantibody measurements by bead-based multiplex serology.
- Table S3. Diagnostic performance of autoantibodies against 64 antigens.
- Table S4. Sensitivity analysis: Diagnostic performance of autoantibodies against 64 antigens (training and test set swapped).
- Table S5. Sensitivity analysis: Diagnostic performance of the top 11 5-marker combinations (training and test set swapped).
- Table S6. Performance of the 5-marker combination anti-MAGEA4 + anti-CTAG1 + anti-TP53 + anti-SDCCAG8 + anti-ERBB2_C in subgroups of gastric cancer patients from DACHSplus, ESTHER II and VERDI and healthy controls.
- Table S7. Dates of diagnosis and blood withdrawal for ESTHER I participants with a diagnosis of gastric cancer during follow-up and corresponding test results of the 5-marker combination anti-MAGEA4 + anti-CTAG1 + anti-TP53 + anti-SDCCAG8 + anti-ERBB2_C.

1. References
2. R code

**a) Supplementary methods**

**Method S1: Data collection**

Clinical data was extracted from hospital records by experienced research assistants who were blind to the blood test results. In addition, standardized patient questionnaires providing information on sociodemographic characteristics, medical history, health status, family history, and lifestyle factors were used.

**Method S2: Classification of cancer and chronic atrophic gastritis**

Gastric and esophageal cancer was classified into stages 0-IV according to the UICC (Union for International Cancer Control) TNM (tumor-node-metastasis) stage classification. Early stage cancers were defined as UICC stage 0-II and late stage cancers were defined as UICC stage III-IV. Patients with adenocarcinoma of the esophagogastric junction (ICD-10 code C16.0) were considered as gastric cancer patients.

To determine the presence of chronic atrophic gastritis in ESTHER I participants, serum concentrations of pepsinogen I (PG I) and pepsinogen II (PG II) were measured by ELISA (Biohit, Helsinki, Finland) in a central laboratory as described previously ^1^. Persons with PG I <70 ng/ml and PG I/PG II <3 were considered as chronic atrophic gastritis patients.

**Method S3: Sensitivity analysis for the assessment of the ability of the best performing 5-marker panel to discriminate gastric cancer cases and controls independent of differences in the age and sex distributions**

In order to correct for age and sex differences, cases and controls of the validation set were first matched according to age (grouped in 5-year intervals) and sex. Cases younger than 50 years or older than 75 years were discarded for this analysis because of unavailability of controls in those age groups. Next we assigned an adjustment weight to each control of the validation set (weight = percentage of cases in corresponding group / percentage of controls in corresponding group). So controls in underrepresented groups (i.e., old males) received a weight larger than 1, and those in overrepresented groups (i.e., young females) received a weight smaller than 1. For calculation of specificity in the age and sex matched control group, the product of the adjustment weight and the 5-marker panel test result (1 for positive result, 0 for negative result) was used.

**b) Supplementary tables**

Table S1. Overview studies.

| **Study (Acronym)** | DACHSplus | BliTz | ESTHER I | ESTHER II | | VERDI |
| --- | --- | --- | --- | --- | --- | --- |
| **German full name** | DACHS = Darmkrebs: Chancen der Verhütung durch Screening | Begleitende Evaluierung innovativer Testverfahren zur Darmkrebs-Früherkennung | Epidemiologische Studie zu Chancen der Verhütung, Früherkennung und optimierten THerapie chronischer ERkrankungen in der älteren Bevölkerung | | | VERlauf der diagnostischen Abklärung bei Krebs-erkrankungen |
| **Study design** | cohort of patients with selected cancers | prospective screening study | population-based cohort study | | patient cohort study | patient cohort study |
| **Location** | Southwestern Germany | Southwestern Germany | Saarland, Germany | | Saarland, Germany | Saarland, Germany |
| **Permit numbers ethics committees** | HD: S-310/2001  BW: M-198-02  RP: 837.419.02 (3637) | HD: S-178/2005  BW: M-118-05-f  RP: 837.047.06 (5145)  SL: 217/13 | HD: S-058/2000  SL: 67/00 | | HD: S-274/2000  RP: 837.383.00 (2749)  SL: 134/00 | HD: S-103/2003  SL: 53/96 |
| **Baseline recruitment** | 2007-ongoing  (-2013 for current study) | 2005-ongoing  (-2011 for  current study) | 2000-2002  (Follow-up:  2000-2011) | | 2001-2003 | 1996-1998 |
| **Place and time of blood sampling** | in hospitals, before surgery | in gastroenterologypractices, before colonoscopy | in general practices at health check-up  + 5 year follow-up blood samples | | in hospitals or at patient’s homes, before or after surgery | |
| **Participants selected for measurements** | GC (n=163)  EC (n=35) | controls (n=235) | controls (n=100)  CAG (n=100)  GC during FU (n=29) | | GC (n=85) | GC (n=68) |
| **Inclusion criteria for current study** | first diagnosis of GC/EC,  blood sample provided | participants of screening colonoscopy,  blood sample provided | no serologically defined CAG*/ serologically defined CAG*/ GC diagnosis during the FU period,  blood sample provided | | first diagnosis of GC,  blood sample provided | first diagnosis of GC,  blood sample provided |
| **Exclusion criteria for current study** | Blood sampling after surgery | Neoplasms found at screening colonoscopy or questionable colonoscopy result, history of CRC or IBD, blood sampling after colonoscopy | History of cancer | | - | - |
| **References** | 2-4 | 5-7 | 8 | | 1 | 1,9 |

Abbreviations: BW = physicians’ board of Baden-Württemberg, CAG = chronic atrophic gastritis, CRC = colorectal cancer, EC = esophageal cancer, FU = follow-up, GC = gastric cancer, HD = Medical Faculty Heidelberg, IBD = inflammatory disease, n = number, RP = physicians’ board of Rheinland-Pfalz, SL = physicians’ board of Saarland.

* Pepsinogen I <70 ng/ml and pepsinogen I/pepsinogen II < 3.

**Table S2. Symbols, official and alternative names of tumor-associated antigens used for autoantibody measurements by bead-based multiplex serology.**

| **Symbol** | **Official protein name** | **Important alternative protein names and abbreviations** | **Length (amino acids)** |
| --- | --- | --- | --- |
| ACRBP | Acrosin-binding protein |  | full length |
| AIMP1 | Aminoacyl tRNA synthase complex-interacting multifunctional protein 1 |  | full length |
| ANXA4 | Annexin A4 |  | full length |
| BIRC5 | Baculoviral IAP repeat-containing protein 5 | survivin | full length |
| CALU | Calumenin |  | full length |
| CAMEL | CTL-recognized antigen on melanoma |  | full length |
| CCNB1 | G2/mitotic-specific cyclin-B1 |  | full length |
| CCND1 | G1/S-specific cyclin-D1 |  | full length |
| CDKN2A | Cyclin-dependent kinase inhibitor 2A | p16-INK4a, p16 | full length |
| CEACAM5 | Carcinoembryonic antigen-related cell adhesion molecule 5 | CEA | full length |
| CT47A | Cancer/testis antigen 47A |  | full length |
| CTAG1 | Cancer/testis antigen 1 | NY-ESO-1 | full length |
| CTAG2 | Cancer/testis antigen 2 |  | full length |
| DCT | L-dopachrome tautomerase | TRP-2 | full length |
| DDX53 | Probable ATP-dependent RNA helicase DDX53 |  | full length |
| EGFR_C | Epidermal growth factor receptor |  | 669-1210 |
| ERBB2_C | Receptor tyrosine-protein kinase erbB-2 | HER2, NEU | 676-1255 |
| ERBB2_N | Receptor tyrosine-protein kinase erbB-2 | HER2, NEU | 23-652 |
| FOLH1_iso1 | Glutamate carboxypeptidase 2 (isoform 1) |  | full length |
| FOLH1_iso7 | Glutamate carboxypeptidase 2 (Isoform 7) |  | full length |
| GAGE7 | G antigen 7 |  | full length |
| GRINA | Protein lifeguard 1 |  | full length |
| GRINA_N | Protein lifeguard 1 |  | 1-164 |
| HIST1H2B | Histone H2B type 1-C/E/F/G/I |  | full length |
| HMGN3 | High mobility group nucleosome-binding domain-containing protein 3 |  | full length |
| HSPA2 | Heat shock-related 70 kDa protein 2 |  | full length |
| HSPA5 | 78 kDa glucose-regulated protein |  | 19-end |
| IGF2BP1 | Insulin-like growth factor 2 mRNA-binding protein 1 | IMP1 | full length |
| IGF2BP3 | Insulin-like growth factor 2 mRNA-binding protein 3 | KOC | full length |
| IMPDH2 | Inosine-5'-monophosphate dehydrogenase 2 |  | full length |
| KLK3_iso1 | Prostate-specific antigen (isoform 1) | PSA | full length |
| KLK3_iso2 | Prostate-specific antigen (isoform 2) | PSA | full length |
| KRAS | GTPase Kras |  | 1-37 |
| MAGEA1 | Melanoma-associated antigen 1 |  | full length |
| MAGEA3 | Melanoma-associated antigen 3 |  | full length |

**Continued on the following page**

**Table S2. Symbols, official and alternative names of tumor-associated antigens used for autoantibody measurements by bead-based multiplex serology (continued).**

| **Symbol** | **Official protein name** | **Important alternative protein names and abbreviations** | **Length (amino acids)** |
| --- | --- | --- | --- |
| MAGEA4 | Melanoma-associated antigen 4 |  | full length |
| MAPKAPK3 | MAP kinase-activated protein kinase 3 |  | full length |
| MDM2 | E3 ubiquitin-protein ligase Mdm2 |  | full length |
| MIA | Melanoma-derived growth regulatory protein |  | full length |
| MLANA | Melanoma antigen recognized by T-cells 1 |  | full length |
| MPHOSPH6 | M-phase phosphoprotein 6 |  | full length |
| MTDH | Protein LYRIC | AEG-1 | 271-451 |
| MUC1_iso8 | Mucin-1 (isoform 8) |  | full length |
| MYC | Myc proto-oncogene protein |  | full length |
| PMEL | Melanocyte protein PMEL |  | full length |
| PSCA | Prostate stem cell antigen |  | full length |
| REG3A | Regenerating islet-derived protein 3-alpha |  | 27-end |
| RPH3AL | Rab effector Noc2 |  | full length |
| RPL13 | 60S ribosomal protein L13 |  | full length |
| SAG | S-arrestin |  | full length |
| SDCCAG8 | Serologically defined colon cancer antigen 8 |  | 225-537 |
| SEC61B | Protein transport protein Sec61 subunit beta |  | full length |
| SNAP25 | Synaptosomal-associated protein 25 |  | full length |
| SPAG9 | C-Jun-amino-terminal kinase-interacting protein 4 |  | full length |
| SPANXA | Sperm protein associated with the nucleus on the X chromosome A |  | full length |
| SSX2 | Protein SSX2 |  | full length |
| SSX4 | Protein SSX4 |  | full length |
| TP53 | Cellular tumor antigen p53 | p53 | full length |
| TPM3_iso1 | Tropomyosin alpha-3 chain (isoform 1) |  | full length |
| TPM3_iso3 | Tropomyosin alpha-3 chain (isoform 3) |  | full length |
| UBE2D1 | Ubiquitin-conjugating enzyme E2 D1 |  | full length |
| UBQLN1 | Ubiquilin-1 |  | full length |
| UCHL3 | Ubiquitin carboxyl-terminal hydrolase isozyme L3 |  | full length |
| VIL1 | Villin-1 |  | full length |

Table S3. Diagnostic performance of autoantibodies against 64 antigens.

| **Antigen** | **Cutoff at 98% specificity (MFI)** | **Training set (controls BliTz, gastric cancer DACHSplus)** | | | | | **Validation set (controls ESTHER I, gastric cancer ESTHER II & VERDI)** | | | | | | |
| --- | --- | --- | --- | --- | --- | --- | --- | --- | --- | --- | --- | --- | --- |
|  |  | **specificity [95% CI] in %** | **sensitivity [95% CI] in %** | | | **mean MFI of all cases with MFIs over cutoff** | **Youden’s index*** | **specificity [95% CI] in %** | **sensitivity [95% CI] in %** | | | | |
|  |  |  | **all GC** | **early stage** | **late stage** |  |  |  | **all GC** | | **early stage** | | **late stage** |
| **MAGEA4**** | 200 | 98 [95-99] | 12 [7-18] | 8 [3-17] | 18 [10-28] | 2886 [14.4 x cutoff] | 0.09 | 95 [88-98] | 14 [10-21] | 10 [4-21] | | 21 [13-32] | |
| **CTAG1**** | 181 | 98 [95-99] | 11 [7-17] | 8 [3-17] | 15 [8-25] | 5108 [28.3 x cutoff] | 0.07 | 98 [93-99] | 9 [5-15] | 10 [4-21] | | 10 [5-20] | |
| **CTAG2** | 211 | 98 [95-99] | 8 [4-13] | 3 [1-11] | 15 [8-25] | 5059 [24 x cutoff] | 0.05 | 98 [93-99] | 8 [4-13] | 6 [2-16] | | 10 [5-20] | |
| **DDX53** | 398 | 98 [95-99] | 8 [4-13] | 11 [5-21] | 7 [3-16] | 4610 [11.6 x cutoff] | 0.05 | 99 [94-100] | 6 [3-11] | 4 [1-13] | | 9 [4-18] | |
| **TP53**** | 318 | 98 [95-99] | 7 [4-12] | 5 [2-13] | 10 [5-20] | 2571 [8.1 x cutoff] | 0.07 | 99 [94-100] | 8 [4-13] | 12 [6-23] | | 7 [3-16] | |
| **MAGEA3** | 341 | 98 [95-99] | 7 [4-12] | 3 [1-11] | 13 [7-23] | 6456 [18.9 x cutoff] | 0.10 | 100 [96-100] | 10 [6-16] | 10 [4-21] | | 13 [7-23] | |
| **SDCCAG8**** | 778 | 98 [95-99] | 6 [3-11] | 8 [3-17] | 4 [2-12] | 2251 [2.9 x cutoff] | 0.02 | 97 [91-99] | 5 [2-10] | 2 [0-10] | | 4 [2-12] | |
| **KLK3_iso2** | 52 | 98 [95-99] | 6 [3-11] | 5 [2-13] | 7 [3-16] | 105 [2 x cutoff] | 0.03 | 98 [93-99] | 5 [2-10] | 2 [0-10] | | 7 [3-16] | |
| **ERBB2_N** | 83 | 98 [95-99] | 5 [3-10] | 5 [2-13] | 6 [2-14] | 222 [2.7 x cutoff] | 0.02 | 95 [88-98] | 7 [4-12] | 4 [1-13] | | 7 [3-16] | |
| **ERBB2_C**** | 175 | 98 [95-99] | 5 [3-10] | 5 [2-13] | 3 [1-10] | 447 [2.6 x cutoff] | 0.02 | 98 [93-99] | 4 [2-9] | 6 [2-16] | | 1 [0-8] | |
| **IGF2BP1** | 237 | 98 [95-99] | 5 [3-10] | 5 [2-13] | 6 [2-14] | 2672 [11.3 x cutoff] | 0.01 | 94 [87-97] | 7 [4-12] | 6 [2-16] | | 9 [4-18] | |
| **GRINA** | 50*** | 98 [95-99] | 5 [3-10] | 5 [2-13] | 6 [2-14] | 72 [1.4 x cutoff] | 0.03 | 98 [93-99] | 5 [3-10] | 4 [1-13] | | 7 [3-16] | |
| **UBQLN1** | 434 | 98 [95-99] | 5 [3-10] | 3 [1-11] | 9 [4-18] | 4202 [9.7 x cutoff] | 0.02 | 97 [91-99] | 5 [2-10] | 4 [1-13] | | 6 [2-14] | |
| **MAGEA1** | 513 | 98 [95-99] | 5 [2-9] | 2 [0-8] | 9 [4-18] | 5177 [10.1 x cutoff] | 0.06 | 99 [94-100] | 7 [4-12] | 4 [1-13] | | 9 [4-18] | |
| **PMEL** | 78 | 98 [95-99] | 4 [2-8] | 3 [1-11] | 4 [2-12] | 510 [6.5 x cutoff] | 0.04 | 99 [94-100] | 5 [2-10] | 4 [1-13] | | 6 [2-14] | |
| **TPM3_iso1** | 657 | 98 [95-99] | 4 [2-8] | 2 [0-8] | 7 [3-16] | 1180 [1.8 x cutoff] | 0.01 | 97 [91-99] | 4 [2-9] | 6 [2-16] | | 4 [2-12] | |
| **MIA** | 52 | 98 [95-99] | 4 [2-8] | 3 [1-11] | 4 [2-12] | 133 [2.6 x cutoff] | 0.04 | 96 [90-98] | 8 [5-14] | 6 [2-16] | | 9 [4-18] | |
| **KLK3_iso1** | 50*** | 100 [98-100] | 4 [2-8] | 2 [0-8] | 6 [2-14] | 80 [1.6 x cutoff] | 0.04 | 99 [94-100] | 5 [2-10] | 2 [0-10] | | 7 [3-16] | |
| **ANXA4** | 50*** | 98 [95-99] | 3 [1-7] | 5 [2-13] | 1 [0-8] | 100 [2 x cutoff] | 0.02 | 97 [91-99] | 5 [3-10] | 2 [0-10] | | 7 [3-16] | |
| **HSPA2** | 417 | 98 [95-99] | 3 [1-7] | 3 [1-11] | 4 [2-12] | 1877 [4.5 x cutoff] | -0.02 | 97 [91-99] | 1 [0-5] | 0 [0-7] | | 1 [0-8] | |
| **TPM3_iso3** | 1282 | 98 [95-99] | 3 [1-7] | 5 [2-13] | 3 [1-10] | 3241 [2.5 x cutoff] | 0.01 | 97 [91-99] | 4 [2-9] | 6 [2-16] | | 4 [2-12] | |
| **MPHOSPH6** | 2301 | 98 [95-99] | 3 [1-7] | 3 [1-11] | 3 [1-10] | 4612 [2 x cutoff] | 0.01 | 98 [93-99] | 3 [1-8] | 2 [0-10] | | 4 [2-12] | |
| **AIMP1** | 493 | 98 [95-99] | 3 [1-7] | 3 [1-11] | 3 [1-10] | 1105 [2.2 x cutoff] | 0.03 | 98 [93-99] | 5 [2-10] | 4 [1-13] | | 4 [2-12] | |
| **MTDH** | 440 | 98 [95-99] | 3 [1-6] | 0 [0-6] | 3 [1-10] | 863 [2 x cutoff] | -0.01 | 97 [91-99] | 2 [1-6] | 4 [1-13] | | 0 [0-5] | |
| **EGFR_C** | 120 | 98 [95-99] | 3 [1-6] | 2 [0-8] | 3 [1-10] | 408 [3.4 x cutoff] | 0.00 | 99 [94-100] | 1 [0-5] | 2 [0-10] | | 0 [0-5] | |
| **GAGE7** | 1665 | 98 [95-99] | 3 [1-6] | 5 [2-13] | 1 [0-8] | 2923 [1.8 x cutoff] | 0.01 | 100 [96-100] | 1 [0-4] | 0 [0-7] | | 1 [0-8] | |
| **IMPDH2** | 56 | 98 [95-99] | 3 [1-6] | 3 [1-11] | 3 [1-10] | 129 [2.3 x cutoff] | -0.03 | 95 [88-98] | 2 [1-6] | 2 [0-10] | | 1 [0-8] | |
| **SPAG9** | 880 | 98 [95-99] | 3 [1-6] | 2 [0-8] | 4 [2-12] | 1011 [1.1 x cutoff] | -0.04 | 93 [86-96] | 3 [1-8] | 4 [1-13] | | 3 [1-10] | |
| **SPANXA** | 3633 | 98 [95-99] | 3 [1-6] | 3 [1-11] | 3 [1-10] | 6561 [1.8 x cutoff] | 0.01 | 100 [96-100] | 1 [0-4] | 0 [0-7] | | 1 [0-8] | |
| **BIRC5** | 813 | 98 [95-99] | 3 [1-6] | 3 [1-11] | 3 [1-10] | 2759 [3.4 x cutoff] | -0.04 | 95 [88-98] | 1 [0-5] | 0 [0-7] | | 1 [0-8] | |
| **SAG** | 872 | 98 [95-99] | 2 [1-6] | 0 [0-6] | 1 [0-8] | 2858 [3.3 x cutoff] | 0.00 | 99 [94-100] | 1 [0-5] | 0 [0-7] | | 3 [1-10] | |
| **CALU** | 981 | 98 [95-99] | 2 [1-6] | 0 [0-6] | 3 [1-10] | 1545 [1.6 x cutoff] | -0.04 | 93 [86-96] | 3 [1-7] | 2 [0-10] | | 1 [0-8] | |

**Continued on following page**

Table S3. Diagnostic performance of autoantibodies against 64 antigens (continued).

| **Antigen** | **Cutoff at 98% specificity (MFI)** | **Training set (controls BliTz, gastric cancer DACHSplus)** | | | | | **Validation set (controls ESTHER I, gastric cancer ESTHER II & VERDI)** | | | | | | |
| --- | --- | --- | --- | --- | --- | --- | --- | --- | --- | --- | --- | --- | --- |
|  |  | **specificity [95% CI] in %** | **sensitivity [95% CI] in %** | | | **mean MFI of all cases with MFIs over cutoff** | **Youden’s index*** | **specificity [95% CI] in %** | **sensitivity [95% CI] in %** | | | | |
|  |  |  | **all GC** | **early stage** | **late stage** |  |  |  | **all GC** | | **early stage** | | **late stage** |
| **CAMEL** | 199 | 98 [95-99] | 2 [1-6] | 2 [0-8] | 3 [1-10] | 747 [3.7 x cutoff] | 0.01 | 99 [94-100] | 2 [1-6] | 2 [0-10] | | 3 [1-10] | |
| **CT47A** | 885 | 98 [95-99] | 2 [1-6] | 2 [0-8] | 3 [1-10] | 1401 [1.6 x cutoff] | 0.00 | 99 [94-100] | 1 [0-4] | 2 [0-10] | | 0 [0-5] | |
| **CCNB1** | 1992 | 98 [95-99] | 2 [1-6] | 2 [0-8] | 1 [0-8] | 5378 [2.7 x cutoff] | -0.02 | 97 [91-99] | 1 [0-4] | 2 [0-10] | | 0 [0-5] | |
| **CCND1** | 102 | 98 [95-99] | 2 [1-6] | 3 [1-11] | 0 [0-5] | 712 [7 x cutoff] | -0.02 | 97 [91-99] | 1 [0-5] | 2 [0-10] | | 1 [0-8] | |
| **IGF2BP3** | 295 | 98 [95-99] | 2 [1-6] | 3 [1-11] | 1 [0-8] | 6272 [21.3 x cutoff] | 0.03 | 96 [90-98] | 7 [4-12] | 6 [2-16] | | 9 [4-18] | |
| **MAPKAPK3** | 268 | 98 [95-99] | 2 [1-6] | 2 [0-8] | 1 [0-8] | 606 [2.3 x cutoff] | 0.00 | 97 [91-99] | 3 [1-8] | 6 [2-16] | | 3 [1-10] | |
| **MUC1_iso8** | 509 | 98 [95-99] | 2 [1-6] | 0 [0-6] | 3 [1-10] | 2216 [4.4 x cutoff] | 0.00 | 97 [91-99] | 3 [1-7] | 0 [0-7] | | 3 [1-10] | |
| **RPL13** | 189 | 98 [95-99] | 2 [1-6] | 3 [1-11] | 0 [0-5] | 815 [4.3 x cutoff] | 0.04 | 97 [91-99] | 7 [4-12] | 8 [3-18] | | 3 [1-10] | |
| **SSX4** | 69 | 98 [95-99] | 2 [1-6] | 3 [1-11] | 1 [0-8] | 80 [1.2 x cutoff] | 0.01 | 97 [91-99] | 4 [2-9] | 4 [1-13] | | 6 [2-14] | |
| **CDKN2A** | 50*** | 99 [97-100] | 1 [0-5] | 2 [0-8] | 1 [0-8] | 387 [7.7 x cutoff] | 0.03 | 100 [96-100] | 3 [1-7] | 4 [1-13] | | 0 [0-5] | |
| **HSPA5** | 50*** | 98 [95-99] | 1 [0-5] | 2 [0-8] | 1 [0-8] | 451 [9 x cutoff] | 0.01 | 100 [96-100] | 1 [0-4] | 2 [0-10] | | 0 [0-5] | |
| **HMGN3** | 1740 | 98 [95-99] | 1 [0-5] | 3 [1-11] | 0 [0-5] | 3105 [1.8 x cutoff] | -0.01 | 97 [91-99] | 2 [1-6] | 2 [0-10] | | 3 [1-10] | |
| **MDM2** | 378 | 98 [95-99] | 1 [0-5] | 0 [0-6] | 1 [0-8] | 1409 [3.7 x cutoff] | 0.01 | 96 [90-98] | 5 [2-10] | 6 [2-16] | | 4 [2-12] | |
| **MLANA** | 664 | 98 [95-99] | 1 [0-5] | 0 [0-6] | 1 [0-8] | 830 [1.3 x cutoff] | 0.02 | 100 [96-100] | 2 [1-6] | 0 [0-7] | | 3 [1-10] | |
| **GRINA_N** | 92 | 98 [95-99] | 1 [0-5] | 2 [0-8] | 1 [0-8] | 428 [4.7 x cutoff] | 0.00 | 97 [91-99] | 3 [1-8] | 2 [0-10] | | 3 [1-10] | |
| **ACRBP** | 1250 | 98 [95-99] | 1 [0-5] | 2 [0-8] | 1 [0-8] | 1740 [1.4 x cutoff] | -0.03 | 96 [90-98] | 1 [0-4] | 2 [0-10] | | 0 [0-5] | |
| **PSCA** | 50*** | 100 [98-100] | 1 [0-5] | 2 [0-8] | 0 [0-5] | 149 [3 x cutoff] | 0.00 | 99 [94-100] | 1 [0-5] | 2 [0-10] | | 0 [0-5] | |
| **FOLH1_iso1** | 50*** | 100 [98-100] | 1 [0-5] | 2 [0-8] | 1 [0-8] | 94 [1.9 x cutoff] | 0.01 | 100 [96-100] | 1 [0-4] | 0 [0-7] | | 1 [0-8] | |
| **FOLH1_iso7** | 50*** | 100 [98-100] | 1 [0-5] | 2 [0-8] | 1 [0-8] | 74 [1.5 x cutoff] | 0.01 | 100 [96-100] | 1 [0-4] | 0 [0-7] | | 1 [0-8] | |
| **REG3A** | 59 | 98 [95-99] | 1 [0-5] | 3 [1-11] | 0 [0-5] | 188 [3.2 x cutoff] | -0.03 | 95 [88-98] | 2 [1-6] | 2 [0-10] | | 3 [1-10] | |
| **SNAP25** | 1185 | 98 [95-99] | 1 [0-5] | 0 [0-6] | 1 [0-8] | 2385 [2 x cutoff] | -0.01 | 98 [93-99] | 1 [0-4] | 0 [0-7] | | 0 [0-5] | |
| **SEC61B** | 50*** | 99 [96-100] | 1 [0-5] | 2 [0-8] | 0 [0-5] | 58 [1.2 x cutoff] | 0.01 | 99 [94-100] | 2 [1-6] | 4 [1-13] | | 0 [0-5] | |
| **UBE2D1** | 344 | 98 [95-99] | 1 [0-5] | 0 [0-6] | 1 [0-8] | 549 [1.6 x cutoff] | 0.02 | 98 [93-99] | 4 [2-9] | 2 [0-10] | | 6 [2-14] | |
| **UCHL3** | 402 | 98 [95-99] | 1 [0-5] | 0 [0-6] | 1 [0-8] | 1520 [3.8 x cutoff] | 0.01 | 100 [96-100] | 1 [0-5] | 2 [0-10] | | 1 [0-8] | |
| **CEACAM5** | 199 | 98 [95-99] | 1 [0-4] | 0 [0-6] | 0 [0-5] | 529 [2.7 x cutoff] | -0.03 | 97 [91-99] | 0 [0-3] | 0 [0-7] | | 0 [0-5] | |
| **MYC** | 188 | 98 [95-99] | 1 [0-4] | 0 [0-6] | 1 [0-8] | 551 [2.9 x cutoff] | -0.03 | 96 [90-98] | 1 [0-4] | 0 [0-7] | | 1 [0-8] | |
| **HIST1H2B** | 2346 | 98 [95-99] | 1 [0-4] | 2 [0-8] | 0 [0-5] | 2634 [1.1 x cutoff] | 0.01 | 100 [96-100] | 1 [0-5] | 2 [0-10] | | 1 [0-8] | |
| **RPH3AL** | 178 | 98 [95-99] | 1 [0-4] | 0 [0-6] | 1 [0-8] | 264 [1.5 x cutoff] | 0.01 | 96 [90-98] | 5 [3-10] | 6 [2-16] | | 4 [2-12] | |
| **SSX2** | 655 | 98 [95-99] | 1 [0-4] | 2 [0-8] | 0 [0-5] | 906 [1.4 x cutoff] | 0.00 | 98 [93-99] | 2 [1-6] | 0 [0-7] | | 3 [1-10] | |
| **DCT** | 50*** | 100 [98-100] | 1 [0-4] | 2 [0-8] | 0 [0-5] | 62 [1.2 x cutoff] | 0.00 | 100 [96-100] | 0 [0-3] | 0 [0-7] | | 0 [0-5] | |
| **VIL1** | 1618 | 98 [95-99] | 1 [0-4] | 0 [0-6] | 1 [0-8] | 3494 [2.2 x cutoff] | 0.01 | 100 [96-100] | 1 [0-4] | 2 [0-10] | | 0 [0-5] | |
| **KRAS** | 50*** | 99 [96-100] | 0 [0-2] | 0 [0-6] | 0 [0-5] | NA [NA] | 0.00 | 99 [94-100] | 1 [0-4] | 0 [0-7] | | 1 [0-8] | |

Abbreviations: MFI = median fluorescence intensity. *Youden’s index (J) = sensitivity + specificity - 1; ** Autoantibody markers selected by algorithm for 5-marker panel; *** The cutoff was set to 50 MFI because the cutoff at 98% specificity was below 50 MFI.

Table S4. Sensitivity analysis: Diagnostic performance of autoantibodies against 64 antigens (training and test set swapped).

| **Antigen** | **Cutoff at 98% specificity (MFI)** | **Training set (controls ESTHER I, gastric cancer ESTHER II & VERDI)** | | | | | **Validation set (controls BliTz, gastric cancer DACHSplus)** | | | | | |
| --- | --- | --- | --- | --- | --- | --- | --- | --- | --- | --- | --- | --- |
|  |  | **specificity [95% CI] in %** | **sensitivity [95% CI] in %** | | | **mean MFI of all cases with MFIs over cutoff** | **Youden’s index*** | **specificity [95% CI] in %** | **sensitivity [95% CI] in %** | | | |
|  |  |  | **all GC** | **early stage** | **late stage** |  |  |  | **all GC** | **early stage** | | **late stage** |
| **MAGEA3** | 103 | 98 [93-99] | 16 [11-23] | 14 [7-26] | 18 [10-28] | 2966 [28.9 x cutoff] | 0.10 | 96 [92-98] | 14 [10-21] | 8 [3-17] | 21 [13-32] | |
| **TP53** | 66 | 98 [93-99] | 14 [10-21] | 25 [16-39] | 12 [6-22] | 2052 [31.3 x cutoff] | 0.07 | 96 [93-98] | 11 [7-17] | 11 [5-21] | 12 [6-22] | |
| **DDX53** | 228 | 98 [93-99] | 10 [6-16] | 6 [2-16] | 16 [9-27] | 3636 [15.9 x cutoff] | 0.07 | 96 [93-98] | 10 [6-16] | 14 [8-25] | 9 [4-18] | |
| **MAGEA4** | 398 | 98 [93-99] | 10 [6-16] | 4 [1-13] | 16 [9-27] | 3306 [8.3 x cutoff] | 0.05 | 98 [95-99] | 7 [4-12] | 5 [2-13] | 12 [6-22] | |
| **CTAG2** | 133 | 98 [93-99] | 9 [5-15] | 10 [4-21] | 10 [5-20] | 4848 [36.3 x cutoff] | 0.06 | 96 [92-98] | 10 [6-16] | 5 [2-13] | 16 [9-27] | |
| **MAGEA1** | 146 | 98 [93-99] | 9 [5-15] | 4 [1-13] | 12 [6-22] | 2403 [16.5 x cutoff] | 0.03 | 95 [91-97] | 8 [5-14] | 3 [1-11] | 16 [9-27] | |
| **CTAG1** | 186 | 98 [93-99] | 9 [5-15] | 10 [4-21] | 10 [5-20] | 5648 [30.3 x cutoff] | 0.09 | 98 [95-99] | 11 [7-17] | 8 [3-17] | 15 [8-25] | |
| **UCHL3** | 74 | 98 [93-99] | 7 [4-12] | 6 [2-16] | 6 [2-14] | 326 [4.4 x cutoff] | -0.07 | 91 [86-94] | 3 [1-6] | 0 [0-6] | 3 [1-10] | |
| **ERBB2_N** | 96 | 98 [93-99] | 6 [3-11] | 2 [0-10] | 7 [3-16] | 121 [1.3 x cutoff] | 0.05 | 100 [98-100] | 5 [3-10] | 5 [2-13] | 6 [2-14] | |
| **ERBB2_C** | 154 | 98 [93-99] | 5 [3-10] | 6 [2-16] | 3 [1-10] | 286 [1.9 x cutoff] | 0.04 | 97 [94-99] | 6 [4-11] | 8 [3-17] | 3 [1-10] | |
| **PMEL** | 53 | 98 [93-99] | 5 [3-10] | 4 [1-13] | 7 [3-16] | 220 [4.1 x cutoff] | 0.02 | 96 [93-98] | 5 [3-10] | 3 [1-11] | 7 [3-16] | |
| **IGF2BP1** | 467 | 98 [93-99] | 5 [3-10] | 4 [1-13] | 7 [3-16] | 3494 [7.5 x cutoff] | 0.01 | 99 [96-100] | 3 [1-6] | 3 [1-11] | 3 [1-10] | |
| **IGF2BP3** | 443 | 98 [93-99] | 5 [3-10] | 4 [1-13] | 7 [3-16] | 2845 [6.4 x cutoff] | 0.01 | 99 [97-100] | 2 [1-6] | 3 [1-11] | 1 [0-8] | |
| **GRINA** | 51 | 98 [93-99] | 5 [3-10] | 4 [1-13] | 7 [3-16] | 118 [2.3 x cutoff] | 0.04 | 99 [96-100] | 5 [3-10] | 5 [2-13] | 6 [2-14] | |
| **ANXA4** | 53 | 98 [93-99] | 5 [2-10] | 2 [0-10] | 6 [2-14] | 90 [1.7 x cutoff] | 0.01 | 99 [96-100] | 3 [1-6] | 3 [1-11] | 1 [0-8] | |
| **SAG** | 374 | 98 [93-99] | 5 [2-10] | 4 [1-13] | 6 [2-14] | 829 [2.2 x cutoff] | 0.00 | 94 [90-96] | 6 [3-11] | 6 [2-15] | 4 [2-12] | |
| **GAGE7** | 565 | 98 [93-99] | 5 [2-10] | 4 [1-13] | 4 [2-12] | 1965 [3.5 x cutoff] | 0.00 | 96 [93-98] | 5 [2-9] | 6 [2-15] | 3 [1-10] | |
| **KLK3_iso1** | 50** | 99 [94-100] | 5 [2-10] | 2 [0-10] | 7 [3-16] | 73 [1.5 x cutoff] | 0.03 | 100 [98-100] | 4 [2-8] | 2 [0-8] | 6 [2-14] | |
| **KLK3_iso2** | 50** | 98 [93-99] | 5 [2-10] | 2 [0-10] | 7 [3-16] | 128 [2.6 x cutoff] | 0.03 | 97 [94-98] | 6 [3-11] | 5 [2-13] | 7 [3-16] | |
| **AIMP1** | 438 | 98 [93-99] | 5 [2-10] | 4 [1-13] | 4 [2-12] | 696 [1.6 x cutoff] | 0.01 | 98 [95-99] | 3 [1-7] | 3 [1-11] | 3 [1-10] | |
| **CAMEL** | 86 | 98 [93-99] | 4 [2-9] | 6 [2-16] | 3 [1-10] | 567 [6.6 x cutoff] | 0.00 | 96 [93-98] | 3 [1-7] | 3 [1-11] | 3 [1-10] | |
| **SDCCAG8** | 795 | 98 [93-99] | 4 [2-9] | 2 [0-10] | 3 [1-10] | 1952 [2.5 x cutoff] | 0.04 | 98 [95-99] | 6 [3-11] | 8 [3-17] | 4 [2-12] | |
| **CT47A** | 567 | 98 [93-99] | 4 [2-9] | 8 [3-18] | 3 [1-10] | 1031 [1.8 x cutoff] | 0.02 | 97 [94-99] | 5 [2-9] | 5 [2-13] | 4 [2-12] | |
| **RPL13** | 224 | 98 [93-99] | 4 [2-9] | 4 [1-13] | 1 [0-8] | 307 [1.4 x cutoff] | 0.00 | 98 [95-99] | 2 [1-6] | 3 [1-11] | 0 [0-5] | |
| **SSX4** | 84 | 98 [93-99] | 4 [2-9] | 4 [1-13] | 6 [2-14] | 316 [3.8 x cutoff] | -0.01 | 98 [95-99] | 1 [0-4] | 2 [0-8] | 0 [0-5] | |
| **UBE2D1** | 337 | 98 [93-99] | 4 [2-9] | 2 [0-10] | 6 [2-14] | 789 [2.3 x cutoff] | -0.01 | 98 [95-99] | 1 [0-5] | 0 [0-6] | 1 [0-8] | |
| **UBQLN1** | 610 | 98 [93-99] | 4 [2-9] | 4 [1-13] | 4 [2-12] | 3486 [5.7 x cutoff] | 0.04 | 99 [96-100] | 5 [3-10] | 3 [1-11] | 9 [4-18] | |
| **HIST1H2B** | 1389 | 98 [93-99] | 3 [1-8] | 4 [1-13] | 3 [1-10] | 2370 [1.7 x cutoff] | -0.02 | 96 [93-98] | 2 [1-6] | 2 [0-8] | 3 [1-10] | |
| **TPM3_iso3** | 1858 | 98 [93-99] | 3 [1-8] | 6 [2-16] | 3 [1-10] | 3772 [2 x cutoff] | 0.01 | 99 [96-100] | 2 [1-6] | 3 [1-11] | 1 [0-8] | |
| **MPHOSPH6** | 2272 | 98 [93-99] | 3 [1-8] | 2 [0-10] | 4 [2-12] | 6711 [3 x cutoff] | 0.01 | 98 [95-99] | 3 [1-7] | 3 [1-11] | 3 [1-10] | |
| **GRINA_N** | 109 | 98 [93-99] | 3 [1-8] | 2 [0-10] | 3 [1-10] | 296 [2.7 x cutoff] | -0.02 | 98 [95-99] | 1 [0-4] | 2 [0-8] | 0 [0-5] | |
| **SPANXA** | 1425 | 98 [93-99] | 3 [1-8] | 0 [0-7] | 7 [3-16] | 2529 [1.8 x cutoff] | 0.02 | 96 [92-98] | 6 [4-11] | 6 [2-15] | 7 [3-16] | |

**Continued on following page**

Table S4. Sensitivity analysis: Diagnostic performance of autoantibodies against 64 antigens (training and test set swapped) (continued).

| **Antigen** | **Cutoff at 98% specificity (MFI)** | **Training set (controls ESTHER I, gastric cancer ESTHER II & VERDI)** | | | | | **Validation set (controls BliTz, gastric cancer DACHSplus)** | | | | | |
| --- | --- | --- | --- | --- | --- | --- | --- | --- | --- | --- | --- | --- |
|  |  | **specificity [95% CI] in %** | **sensitivity [95% CI] in %** | | | **mean MFI of all cases with MFIs over cutoff** | **Youden’s index*** | **specificity [95% CI] in %** | **sensitivity [95% CI] in %** | | | |
|  |  |  | **all GC** | **early stage** | **late stage** |  |  |  | **all GC** | **early stage** | | **late stage** |
| **CDKN2A** | 50** | 100 [96-100] | 3 [1-7] | 4 [1-13] | 0 [0-5] | 1704 [34.1 x cutoff] | 0.00 | 99 [97-100] | 1 [0-5] | 2 [0-8] | 1 [0-8] | |
| **TPM3_iso1** | 949 | 98 [93-99] | 3 [1-7] | 6 [2-16] | 1 [0-8] | 1781 [1.9 x cutoff] | 0.01 | 100 [98-100] | 2 [1-6] | 2 [0-8] | 3 [1-10] | |
| **MIA** | 79 | 98 [93-99] | 3 [1-7] | 2 [0-10] | 4 [2-12] | 102 [1.3 x cutoff] | 0.01 | 100 [98-100] | 2 [1-6] | 2 [0-8] | 1 [0-8] | |
| **SNAP25** | 948 | 98 [93-99] | 3 [1-7] | 2 [0-10] | 1 [0-8] | 1165 [1.2 x cutoff] | 0.00 | 97 [94-99] | 3 [1-6] | 0 [0-6] | 4 [2-12] | |
| **SSX2** | 494 | 98 [93-99] | 3 [1-7] | 0 [0-7] | 4 [2-12] | 1177 [2.4 x cutoff] | -0.02 | 97 [94-99] | 1 [0-4] | 2 [0-8] | 0 [0-5] | |
| **VIL1** | 745 | 98 [93-99] | 3 [1-7] | 4 [1-13] | 1 [0-8] | 2355 [3.2 x cutoff] | -0.02 | 96 [92-98] | 3 [1-6] | 2 [0-8] | 4 [2-12] | |
| **MTDH** | 470 | 98 [93-99] | 2 [1-6] | 4 [1-13] | 0 [0-5] | 844 [1.8 x cutoff] | 0.01 | 99 [96-100] | 3 [1-6] | 0 [0-6] | 3 [1-10] | |
| **IMPDH2** | 61 | 98 [93-99] | 2 [1-6] | 2 [0-10] | 1 [0-8] | 166 [2.7 x cutoff] | 0.00 | 98 [95-99] | 2 [1-6] | 2 [0-8] | 3 [1-10] | |
| **MLANA** | 298 | 98 [93-99] | 2 [1-6] | 0 [0-7] | 3 [1-10] | 1772 [5.9 x cutoff] | 0.03 | 97 [94-98] | 6 [3-11] | 3 [1-11] | 6 [2-14] | |
| **MUC1_iso8** | 631 | 98 [93-99] | 2 [1-6] | 0 [0-7] | 1 [0-8] | 1346 [2.1 x cutoff] | 0.00 | 98 [95-99] | 1 [0-5] | 0 [0-6] | 1 [0-8] | |
| **REG3A** | 73 | 98 [93-99] | 2 [1-6] | 2 [0-10] | 3 [1-10] | 98 [1.3 x cutoff] | 0.00 | 100 [98-100] | 1 [0-4] | 2 [0-8] | 0 [0-5] | |
| **SEC61B** | 50** | 99 [94-100] | 2 [1-6] | 4 [1-13] | 0 [0-5] | 77 [1.5 x cutoff] | 0.00 | 99 [96-100] | 1 [0-5] | 2 [0-8] | 0 [0-5] | |
| **EGFR_C** | 114 | 98 [93-99] | 1 [0-5] | 2 [0-10] | 0 [0-5] | 136 [1.2 x cutoff] | 0.00 | 98 [95-99] | 3 [1-6] | 2 [0-8] | 3 [1-10] | |
| **PSCA** | 50** | 99 [94-100] | 1 [0-5] | 2 [0-10] | 0 [0-5] | 56 [1.1 x cutoff] | 0.01 | 100 [98-100] | 1 [0-5] | 2 [0-8] | 0 [0-5] | |
| **RPH3AL** | 518 | 98 [93-99] | 1 [0-5] | 2 [0-10] | 0 [0-5] | 1093 [2.1 x cutoff] | 0.00 | 100 [98-100] | 0 [0-2] | 0 [0-6] | 0 [0-5] | |
| **CALU** | 2717 | 98 [93-99] | 1 [0-4] | 0 [0-7] | 0 [0-5] | 3052 [1.1 x cutoff] | -0.01 | 99 [97-100] | 0 [0-2] | 0 [0-6] | 0 [0-5] | |
| **MYC** | 211 | 98 [93-99] | 1 [0-4] | 0 [0-7] | 1 [0-8] | 707 [3.3 x cutoff] | -0.01 | 98 [95-99] | 1 [0-4] | 0 [0-6] | 1 [0-8] | |
| **CCND1** | 354 | 98 [93-99] | 1 [0-4] | 2 [0-10] | 0 [0-5] | 618 [1.7 x cutoff] | 0.00 | 100 [98-100] | 1 [0-4] | 2 [0-8] | 0 [0-5] | |
| **HSPA5** | 50** | 100 [96-100] | 1 [0-4] | 2 [0-10] | 0 [0-5] | 691 [13.8 x cutoff] | 0.00 | 98 [95-99] | 1 [0-5] | 2 [0-8] | 1 [0-8] | |
| **HMGN3** | 2728 | 98 [93-99] | 1 [0-4] | 0 [0-7] | 1 [0-8] | 3664 [1.3 x cutoff] | -0.01 | 98 [95-99] | 1 [0-4] | 2 [0-8] | 0 [0-5] | |
| **HSPA2** | 1337 | 98 [93-99] | 1 [0-4] | 0 [0-7] | 0 [0-5] | 2475 [1.9 x cutoff] | 0.00 | 99 [97-100] | 1 [0-5] | 2 [0-8] | 1 [0-8] | |
| **MAPKAPK3** | 598 | 98 [93-99] | 1 [0-4] | 0 [0-7] | 1 [0-8] | 2445 [4.1 x cutoff] | 0.00 | 100 [98-100] | 1 [0-4] | 0 [0-6] | 0 [0-5] | |
| **MDM2** | 682 | 98 [93-99] | 1 [0-4] | 0 [0-7] | 1 [0-8] | 1046 [1.5 x cutoff] | 0.01 | 100 [98-100] | 1 [0-5] | 0 [0-6] | 1 [0-8] | |
| **ACRBP** | 1711 | 98 [93-99] | 1 [0-4] | 2 [0-10] | 0 [0-5] | 2522 [1.5 x cutoff] | -0.01 | 98 [95-99] | 1 [0-4] | 0 [0-6] | 1 [0-8] | |
| **FOLH1_iso1** | 50** | 100 [96-100] | 1 [0-4] | 0 [0-7] | 1 [0-8] | 65 [1.3 x cutoff] | 0.01 | 100 [98-100] | 1 [0-5] | 2 [0-8] | 1 [0-8] | |
| **FOLH1_iso7** | 50** | 100 [96-100] | 1 [0-4] | 0 [0-7] | 1 [0-8] | 65 [1.3 x cutoff] | 0.01 | 100 [98-100] | 1 [0-5] | 2 [0-8] | 1 [0-8] | |
| **KRAS** | 50** | 99 [94-100] | 1 [0-4] | 0 [0-7] | 1 [0-8] | 266 [5.3 x cutoff] | -0.01 | 99 [96-100] | 0 [0-2] | 0 [0-6] | 0 [0-5] | |
| **CEACAM5** | 222 | 98 [93-99] | 0 [0-3] | 0 [0-7] | 0 [0-5] | NA | -0.02 | 98 [95-99] | 1 [0-4] | 0 [0-6] | 0 [0-5] | |
| **CCNB1** | 2393 | 98 [93-99] | 0 [0-3] | 0 [0-7] | 0 [0-5] | NA | 0.00 | 99 [96-100] | 1 [0-5] | 2 [0-8] | 1 [0-8] | |
| **SPAG9** | 4451 | 98 [93-99] | 0 [0-3] | 0 [0-7] | 0 [0-5] | NA | -0.01 | 99 [97-100] | 0 [0-2] | 0 [0-6] | 0 [0-5] | |
| **BIRC5** | 1504 | 98 [93-99] | 0 [0-3] | 0 [0-7] | 0 [0-5] | NA | 0.02 | 100 [98-100] | 3 [1-6] | 3 [1-11] | 3 [1-10] | |
| **DCT** | 50** | 100 [96-100] | 0 [0-3] | 0 [0-7] | 0 [0-5] | NA | 0.01 | 100 [98-100] | 1 [0-4] | 2 [0-8] | 0 [0-5] | |

Abbreviations: MFI = median fluorescence intensity. *Youden’s index (J) = sensitivity + specificity - 1; ** The cutoff was set to 50 MFI because the cutoff at 98% specificity was below 50 MFI.

Table S5. Sensitivity analysis: Diagnostic performance of the top 11 5-marker combinations* (training and test set swapped).

| **No.** | **Autoantibodies** | **Training set** | | | **Validation set** | | |
| --- | --- | --- | --- | --- | --- | --- | --- |
|  |  | **Youden’s index** | **sensitivity [95% CI]**  **in %** | **specificity [95% CI]**  **in %** | **Youden’s index** | **sensitivity [95% CI]**  **in %** | **specificity [95% CI]**  **in %** |
| **1** | anti-MAGEA3 + anti-TP53 + anti-DDX53  + anti-UCHL3 + anti-CTAG1 | 0.34 | 43 (35-51) | 91 (83-95) | 0.17 | 37 (30-45) | 80 (74-85) |
| **2** | anti-MAGEA3 + anti-TP53 + anti-DDX53  + anti-CAMEL + anti-UCHL3 | 0.33 | 42 (35-51) | 91 (83-95) | 0.13 | 34 (27-41) | 79 (73-84) |
| **3** | anti-MAGEA3 + anti-TP53 + anti-DDX53  + anti-CAMEL + anti-CTAG1 | 0.33 | 41 (33-49) | 92 (85-96) | 0.21 | 36 (29-44) | 85 (80-89) |
| **4** | anti-MAGEA3 + anti-TP53 + anti-DDX53  + anti-CTAG1 + anti-AIMP1 | 0.33 | 41 (33-49) | 92 (85-96) | 0.22 | 37 (30-45) | 86 (81-90) |
| **5** | anti-MAGEA3 + anti-TP53 + anti-DDX53  + anti-UCHL3 + anti-ANXA4 | 0.33 | 42 (34-50) | 91 (83-95) | 0.13 | 32 (25-40) | 80 (75-85) |
| **6** | anti-MAGEA3 + anti-TP53 + anti-DDX53  + anti-UCHL3 + anti-AIMP1 | 0.33 | 42 (34-50) | 91 (83-95) | 0.13 | 34 (27-42) | 79 (73-84) |
| **7** | anti-MAGEA3 + anti-TP53 + anti-DDX53  + anti-CAMEL + anti-ERBB2_C | 0.32 | 40 (33-49) | 92 (85-96) | 0.21 | 36 (29-44) | 84 (79-89) |
| **8** | anti-MAGEA3 + anti-TP53 + anti-DDX53  + anti-CAMEL + anti-PMEL | 0.32 | 40 (33-49) | 92 (85-96) | 0.17 | 34 (27-41) | 83 (78-88) |
| **9** | anti-MAGEA3 + anti-TP53 + anti-DDX53  + anti-CAMEL + anti-RPL13 | 0.32 | 40 (33-49) | 92 (85-96) | 0.18 | 32 (25-40) | 85 (80-89) |
| **10** | anti-MAGEA3 + anti-TP53 + anti-DDX53  + anti-CAMEL + anti-AIMP1 | 0.32 | 40 (33-49) | 92 (85-96) | 0.18 | 34 (27-41) | 84 (79-89) |
| **11** | anti-MAGEA3 + anti-TP53 + anti-DDX53  + anti-UCHL3 + anti-CTAG2 | 0.32 | 42 (35-51) | 90 (82-94) | 0.15 | 36 (29-44) | 79 (73-83) |

*The 11 5-marker combinations were selected based on a maximum Youden’s index (J=sensitivity+specificity-1) in the training set. Anti-MAGEA3, anti-TP53 and anti-DDX53 are present in all 11 combinations.

**Table S6. Performance of the 5-marker combination anti-MAGEA4 + anti-CTAG1 + anti-TP53 + anti-SDCCAG8 + anti-ERBB2_C** in subgroups of gastric cancer patients from DACHSplus, ESTHER II and VERDI and healthy controls.

| **Subgroup** | | **Training set (BliTz controls + gastric cancer cases DACHSplus)** | | | | | | **Validation set (ESTHER I controls + gastric cancer cases ESTHER II, VERDI)** | | | | | |
| --- | --- | --- | --- | --- | --- | --- | --- | --- | --- | --- | --- | --- | --- |
|  |  | **n cases** | **n controls** | **sensitivity**  **(95 % CI)** | **specificity**  **(95 % CI)** | **Youden's index** | **p-value*** | **n cases** | **n controls** | **sensitivity**  **(95 % CI)** | **specificity**  **(95 % CI)** | **Youden's index** | **p-value*** |
| **(all samples)** | | 155 | 224 | 34 [27-41] | 89 [85-93] | 0.23 | NA, NA | 146 | 97 | 32 [25-40] | 87 [78-92] | 0.19 | NA, NA |
| **study** | **ESTHER II** |  |  |  |  |  | NA, NA | 81 | 97 | 35 [25-45] | 87 [78-92] | 0.21 | 0.59, 1.00 |
|  | **VERDI** |  |  |  |  |  |  | 65 | 97 | 29 [20-41] | 87 [78-92] | 0.16 |  |
| **UICC stage** | **0-II** | 64 | 224 | 31 [21-43] | 89 [85-93] | 0.21 | 0.58, 1.00 | 51 | 97 | 35 [24-49] | 87 [78-92] | 0.22 | 0.84, 1.00 |
|  | **III-IV** | 68 | 224 | 37 [26-49] | 89 [85-93] | 0.26 |  | 68 | 97 | 32 [22-44] | 87 [78-92] | 0.19 |  |
| **sex** | **men** | 106 | 101 | 30 [22-39] | 89 [82-94] | 0.19 | 0.21, 1.00 | 95 | 49 | 34 [25-44] | 86 [73-93] | 0.19 | 0.85, 1.00 |
|  | **women** | 49 | 123 | 41 [28-55] | 89 [83-94] | 0.30 |  | 49 | 48 | 31 [20-45] | 88 [75-94] | 0.18 |  |
| **age** | **< 65 years** | 72 | 145 | 28 [19-39] | 90 [84-94] | 0.18 | 0.18, 0.50 | 62 | 64 | 24 [15-36] | 89 [79-95] | 0.13 | 0.07, 0.36 |
|  | **≥ 65 years** | 83 | 79 | 39 [29-49] | 87 [78-93] | 0.26 |  | 81 | 33 | 40 [30-50] | 82 [66-91] | 0.21 |  |
| **time of bood withdrawal** | **≤ 2 weeks after surgery** | 146 | 224 | 34 [26-42] | 89 [85-93] | 0.23 | NA, NA | 88 | 97 | 27 [19-37] | 87 [78-92] | 0.14 | 0.32, 1.00 |
|  | **>2 weeks after surgery** |  |  |  |  |  |  | 44 | 97 | 36 [24-51] | 87 [78-92] | 0.23 |  |
| **neo-adjuvant therapy** | **no** | 66 | 224 | 33 [23-45] | 89 [85-93] | 0.23 | 1.00, 1.00 | 127 | 97 | 31 [23-39] | 87 [78-92] | 0.17 | 0.51, 1.00 |
|  | **yes** | 89 | 224 | 34 [25-44] | 89 [85-93] | 0.23 |  | 11 | 97 | 18 [5-48] | 87 [78-92] | 0.05 |  |
| **cancer type** | **non cardia carcinoma** | 54 | 224 | 33 [22-47] | 89 [85-93] | 0.23 | 0.70, 1.00 | 92 | 97 | 29 [21-39] | 87 [78-92] | 0.16 | 0.64, 1.00 |
|  | **cardia carcinoma** | 64 | 224 | 38 [27-50] | 89 [85-93] | 0.27 |  | 28 | 97 | 36 [21-54] | 87 [78-92] | 0.22 |  |
| ***H. pylori* infection status** | ***H. pylori* negative** | 56 | 66 | 36 [24-49] | 91 [82-96] | 0.27 | 0.73, 0.93 | 31 | 47 | 42 [26-59] | 79 [65-88] | 0.21 | 0.40, 0.09 |
|  | ***H. pylori* positive, CagA negative** | 34 | 41 | 35 [21-52] | 88 [74-95] | 0.23 |  | 11 | 27 | 18 [5-48] | 96 [82-100] | 0.14 |  |
|  | ***H. pylori* positive, CagA positive** | 58 | 30 | 29 [19-42] | 90 [74-97] | 0.19 |  | 36 | 23 | 33 [20-50] | 91 [73-98] | 0.25 |  |

*p value for differences in sensitivity and specificity between subgroups (Fisher's exact test)

**Table S7. Dates of diagnosis and blood withdrawal for ESTHER I participants with a diagnosis of gastric cancer during follow-up and corresponding test results of the 5-marker combination anti-MAGEA4 + anti-CTAG1 + anti-TP53 + anti-SDCCAG8 + anti-ERBB2_C.**

| **Patient ID** | **Month of gastric cancer diagnosis** | **Baseline sample** | | | **5-year follow-up sample** | | |
| --- | --- | --- | --- | --- | --- | --- | --- |
|  |  | **Month of bloodwith-drawal** | **Days between blood withdrawal and diagnosis** | **Test result**  **5-marker combi-nation** | **Month of bloodwith-drawal** | **Days between blood withdrawal and diagnosis** | **Test result 5-marker combi-nation** |
| 1 | Mar-2002 | Mar-2002 | 1 | negative | no follow-up sample | | |
| 2 | Mar-2002 | Sep-2001 | 185 | negative | no follow-up sample | | |
| 3 | Mar-2002 | Mar-2001 | 364 | negative | no follow-up sample | | |
| 4 | Feb-2002 | Jan-2001 | 407 | negative | no follow-up sample | | |
| 5 | Jan-2002 | Nov-2000 | 434 | **positive** | no follow-up sample | | |
| 6 | Nov-2002 | Aug-2001 | 465 | negative | Oct-2006 | -1445 | negative |
| 7 | Jan-2003 | Sep-2001 | 483 | negative | no follow-up sample | | |
| 8 | Jul-2003 | Aug-2001 | 678 | negative | Dec-2006 | -1247 | negative |
| 9 | Dec-2003 | Dec-2001 | 727 | negative | no follow-up sample | | |
| 10 | Jul-2003 | Nov-2000 | 960 | **positive** | no follow-up sample | | |
| 11 | Sep-2003 | Aug-2000 | 1128 | negative | no follow-up sample | | |
| 12 | Aug-2004 | Jan-2001 | 1303 | negative | no follow-up sample | | |
| 13 | Mar-2006 | Jan-2002 | 1506 | negative | no follow-up sample | | |
| 14 | Sep-2007 | Feb-2002 | 2024 | negative | Aug-2007 | 33 | negative |
| 15 | Jan-2007 | Oct-2000 | 2267 | negative | Nov-2005 | 428 | **positive** |
| 16 | Feb-2008 | Feb-2001 | 2541 | negative | no follow-up sample | | |
| 17 | Jul-2008 | Feb-2001 | 2702 | negative | Mar-2006 | 846 | **positive** |
| 18 | Nov-2009 | Jan-2002 | 2860 | negative | May-2007 | 926 | negative |
| 19 | Aug-2010 | May-2002 | 3024 | negative | Sep-2007 | 1083 | NA |
| 20 | Nov-2009 | Apr-2001 | 3135 | negative | Apr-2006 | 1322 | **positive** |
| 21 | Jun-2009 | Oct-2000 | 3139 | NA | Oct-2005 | 1325 | negative |
| 22 | Aug-2009 | Dec-2000 | 3184 | negative | Sep-2006 | 1073 | **positive** |
| 23 | Aug-2009 | Oct-2000 | 3214 | **positive** | Sep-2005 | 1429 | negative |
| 24 | Nov-2010 | Nov-2001 | 3294 | negative | Feb-2007 | 1382 | negative |
| 25 | Oct-2010 | Aug-2001 | 3346 | negative | Jan-2007 | 1372 | negative |
| 26 | Jun-2010 | Nov-2000 | 3501 | NA | Oct-2005 | 1723 | NA |
| 27 | Feb-2011 | Jul-2001 | 3525 | **positive** | no follow-up sample | | |
| 28 | Feb-2011 | Oct-2000 | 3781 | negative | no follow-up sample | | |
| 29 | Aug-2011 | Jan-2001 | 3857 | **positive** | no follow-up sample | | |

**c) References**

1 Gao, L., Weck, M. N., Michel, A., Pawlita, M. & Brenner, H. Association between chronic atrophic gastritis and serum antibodies to 15 Helicobacter pylori proteins measured by multiplex serology. *Cancer Res* **69**, 2973-2980 (2009).

2 Brenner, H., Chang-Claude, J., Seiler, C. M., Sturmer, T. & Hoffmeister, M. Does a negative screening colonoscopy ever need to be repeated? *Gut* **55**, 1145-1150 (2006).

3 Brenner, H., Chang-Claude, J., Seiler, C. M., Sturmer, T. & Hoffmeister, M. Case-control study supports extension of surveillance interval after colonoscopic polypectomy to at least 5 yr. *Am J Gastroenterol* **102**, 1739-1744 (2007).

4 Brenner, H., Chang-Claude, J., Seiler, C. M., Rickert, A. & Hoffmeister, M. Protection from colorectal cancer after colonoscopy: a population-based, case-control study. *Ann Intern Med* **154**, 22-30 (2011).

5 Hundt, S., Haug, U. & Brenner, H. Comparative evaluation of immunochemical fecal occult blood tests for colorectal adenoma detection. *Ann Intern Med* **150**, 162-169 (2009).

6 Brenner, H., Tao, S. & Haug, U. Low-dose aspirin use and performance of immunochemical fecal occult blood tests. *JAMA* **304**, 2513-2520 (2010).

7 Haug, U., Hundt, S. & Brenner, H. Quantitative immunochemical fecal occult blood testing for colorectal adenoma detection: evaluation in the target population of screening and comparison with qualitative tests. *Am J Gastroenterol* **105**, 682-690 (2010).

8 Brenner, H., Rothenbacher, D. & Weck, M. N. Epidemiologic findings on serologically defined chronic atrophic gastritis strongly depend on the choice of the cutoff-value. *International Journal of Cancer* **121**, 2782-2786 (2007).

9 Brenner, H., Arndt, V., Stegmaier, C., Ziegler, H. & Rothenbacher, D. Is Helicobacter pylori infection a necessary condition for noncardia gastric cancer? *Am J Epidemiol* **159**, 252-258 (2004).

**d) R code**

###########################################################################

# R code for the evaluation of single autoantibody markers (Table 2,

# Table S3) and marker combinations (Table 3)

# Written by Simone Werner and Hongda Chen

###########################################################################

# R packages

library**(**matrixStats**)**

library**(**parallel**)**

library**(**Hmisc**)**

###########################################################################

# For reasons of data protection original data is not used in this program

# --> generation of mock up data

trainingtest**<-**c**(**rep**(**"training",379**)**,rep**(**"test",243**))**

group**<-**c**(**rep**(**"GC_dp",155**)**,rep**(**"controls_blitz",224**)**,

rep**(**"GC_esther2verdi",146**)**,rep**(**"controls_e1_bl",97**))**

casecontrol**<-**c**(**rep**(**1,155**)**,rep**(**0,224**)**,rep**(**1,146**)**,rep**(**0,97**))**

stage**<-**c**(**sample**(**1**:**4,155, replace**=**T**)**,rep**(NA**,224**)**,sample**(**1**:**4,146, replace**=**T**)**,rep**(NA**,97**))**

MFIs**<-**matrix**(**rnorm**(**39808,100,100**)**,ncol**=**64**)**

data**<-**data.frame**(**trainingtest,group,casecontrol,stage,MFIs**)**

colnames**(**data**)[**5**:**68**]<-**c**(**"TP53", "CDKN2A", "MTDH", "ANXA4", "SAG", "CALU", "CAMEL", "SDCCAG8", "CEACAM5", "MYC", "CT47A", "CTAG2", "CCNB1", "CCND1", "DDX53", "EGFR_C ", "ERBB2_N", "ERBB2_C", "GAGE7", "PMEL", "HSPA5", "HIST1H2B", "HMGN3", "HSPA2", "TPM3_iso1", "TPM3_iso3", "IGF2BP1", "IMPDH2", "IGF2BP3", "MAGEA1", "MAGEA3", "MAGEA4", "MAPKAPK3", "MDM2", "MLANA", "MPHOSPH6", "MIA", "MUC1_iso8", "GRINA", "GRINA_N", "CTAG1", "ACRBP", "KLK3_iso1", "KLK3_iso2", "PSCA", "FOLH1_iso1", "FOLH1_iso7", "KRAS", "REG3A", "RPH3AL", "RPL13", "SNAP25", "AIMP1", "SEC61B", "SPAG9", "SPANXA", "SSX2", "SSX4", "BIRC5", "DCT", "UBE2D1", "UBQLN1", "UCHL3", "VIL1"**)**

View**(**data**)**

# Variables in the moke up data set

# variable 1 ("trainingtest") indicates if a sample belongs to the training # or the test set

# variable 2 ("group") indicates the groups of participants:

# - GC_dp = gastric cancer cases DACHSplus study

# - controls_blitz = controls BliTz study

# - GC_esther2verdi = gastric cancer cases ESTHER II or VERDI study

# - controls_e1_bl = controls ESTHER I study

# variable 3 ("casecontrol") indicates the case-control status (0=control,

# 1=case)

# variable 4 ("stage") indicates the cancer stage

# variables 4-67 represent median fluorescence intensities (MFIs) for the

# 64 autoantibody markers

###########################################################################

# Create binary variables

# split dataset in dataset with ids and dataset with results

ids**<-**data**[**,c**(**1**:**4**)]**

results**<-**as.matrix**(**data**[**,5**:**68**])**

# calculate cutoffs

controls_training**<-**results**[**ids**$**group**==**"controls_blitz",**]**

p98**<-**colQuantiles**(**controls_training, probs**=**0.98, drop**=**F**)**

p98_2**<-**ifelse**(**p98**<**50,50,p98**)**

p98_3**<-**matrix**(**rbind**(**rep**(**p98_2,622**))**,nrow**=**622, byrow**=**T**)**

results2**<-**ifelse**(**results**>**p98_3,1,0**)**

data_binary**<-**cbind**(**ids,results2**)**

View**(**data_binary**)**

###########################################################################

###########################################################################

# Table 2

# calculate sensitivity,specificity, youden's index (all stages)

data_binary_ctrl_train**<-**data_binary**[**data_binary**$**group**==**"controls_blitz",5**:**68**]**

data_binary_gc_train**<-**data_binary**[**data_binary**$**group**==**"GC_dp",5**:**68**]**

data_binary_ctrl_test**<-**data_binary**[**data_binary**$**group**==**"controls_e1_bl",5**:**68**]**

data_binary_gc_test**<-**data_binary**[**data_binary**$**group**==**"GC_esther2verdi",5**:**68**]**

table**(**data_binary**$**group**)**

spe_train**<-**round**((**1**-**colSums**(**data_binary_ctrl_train**)/**224**)***100,2**)**

sen_train**<-**round**(**colSums**(**data_binary_gc_train**)/**155*****100,2**)**

spe_test**<-**round**((**1**-**colSums**(**data_binary_ctrl_test**)/**97**)***100,2**)**

sen_test**<-**round**(**colSums**(**data_binary_gc_test**)/**146*****100,2**)**

youden_train**<-**round**((**1**-**colSums**(**data_binary_ctrl_train**)/**224**)+**colSums**(**data_binary_gc_train**)/**155**-**1,2**)**

youden_test**<-**round**((**1**-**colSums**(**data_binary_ctrl_test**)/**97**)+**colSums**(**data_binary_gc_test**)/**146**-**1,2**)**

ci_sen_train**<-**round**((**binconf**(**colSums**(**data_binary_gc_train**)**,155 **)***100**))**

sen2_train**<-**paste0**(**ci_sen_train**[**,1**]**," [",ci_sen_train**[**,2**]**,"-"

,ci_sen_train**[**,3**]**,"]"**)**

ci_sen_test**<-**round**((**binconf**(**colSums**(**data_binary_gc_test**)**,146**)***100**))**

sen2_test**<-**paste0**(**ci_sen_test**[**,1**]**," [",ci_sen_test**[**,2**]**,"-"

,ci_sen_test**[**,3**]**,"]"**)**

ci_spe_train**<-**round**((**binconf**(**224**-**colSums**(**data_binary_ctrl_train**)**,224**)***100**))**

spe2_train**<-**paste0**(**ci_spe_train**[**,1**]**," [",ci_spe_train**[**,2**]**,"-"

,ci_spe_train**[**,3**]**,"]"**)**

ci_spe_test**<-**round**((**binconf**(**97**-**colSums**(**data_binary_ctrl_test**)**,97**)***100**))**

spe2_test**<-**paste0**(**ci_spe_test**[**,1**]**," [",ci_spe_test**[**,2**]**,"-"

,ci_spe_test**[**,3**]**,"]"**)**

###########################################################################

# calculate sensitivity early and late stages

gc_train012**<-**data_binary**[**data_binary**$**group**==**"GC_dp"**&**data_binary**$**stage %in% c**(**0,1,2**)**,5**:**68**]**

gc_train34**<-**data_binary**[**data_binary**$**group**==**"GC_dp"**&**data_binary**$**stage %in% c**(**3,4**)**,5**:**68**]**

gc_test012**<-**data_binary**[**data_binary**$**group**==**"GC_esther2verdi"

**&**data_binary**$**stage %in% c**(**0,1,2**)**,5**:**68**]**

gc_test34**<-**data_binary**[**data_binary**$**group**==**"GC_esther2verdi"

**&**data_binary**$**stage %in% c**(**3,4**)**,5**:**68**]**

sen_train012**<-**round**(**colSums**(**gc_train012**)/**dim**(**gc_train012**)[**1**]***100,2**)**

sen_train34**<-**round**(**colSums**(**gc_train34**)/**dim**(**gc_train34**)[**1**]***100,2**)**

sen_test012**<-**round**(**colSums**(**gc_test012**)/**dim**(**gc_test012**)[**1**]***100,2**)**

sen_test34**<-**round**(**colSums**(**gc_test34**)/**dim**(**gc_test34**)[**1**]***100,2**)**

ci_sen_train012**<-**round**((**binconf**(**colSums**(**gc_train012**)**,dim**(**gc_train012**)[**1**])***100**))**

sen2_train012**<-**paste0**(**ci_sen_train012**[**,1**]**," [",ci_sen_train012**[**,2**]**,

"-",ci_sen_train012**[**,3**]**,"]"**)**

ci_sen_train34**<-**round**((**binconf**(**colSums**(**gc_train34**)**,dim**(**gc_train34**)[**1**])***100**))**

sen2_train34**<-**paste0**(**ci_sen_train34**[**,1**]**," [",ci_sen_train34**[**,2**]**,

"-",ci_sen_train34**[**,3**]**,"]"**)**

ci_sen_test012**<-**round**((**binconf**(**colSums**(**gc_test012**)**,dim**(**gc_test012**)[**1**])***100**))**

sen2_test012**<-**paste0**(**ci_sen_test012**[**,1**]**," [",ci_sen_test012**[**,2**]**,

"-",ci_sen_test012**[**,3**]**,"]"**)**

ci_sen_test34**<-**round**((**binconf**(**colSums**(**gc_test34**)**,dim**(**gc_test34**)[**1**])***100**))**

sen2_test34**<-**paste0**(**ci_sen_test34**[**,1**]**," [",ci_sen_test34**[**,2**]**,

"-",ci_sen_test34**[**,3**]**,"]"**)**

###########################################################################

# calculate mean fluorescence over cutoff

results3**<-**ifelse**(**results**>**p98_3,results,**NA)**

data_binary2**<-**cbind**(**results3,ids**)**

data_binary2_ctrl_train**<-**data_binary2**[**data_binary2**$**group**==**"controls_blitz",1**:**64**]**

data_binary2_gc_train**<-**data_binary2**[**data_binary2**$**group**==**"GC_dp",1**:**64**]**

data_binary2_ctrl_test**<-**data_binary2**[**data_binary2**$**group**==**"controls_e1_bl",1**:**64**]**

data_binary2_gc_test**<-**data_binary2**[**data_binary2**$**group**==**"GC_esther2verdi",1**:**64**]**

mean_MFIs_gc_train**<-**round**(**colMeans**(**data_binary2_gc_train,na.rm **=**T**))**

mean_MFIs_gc_train2**<-**round**((**colMeans**(**data_binary2_gc_train,

na.rm **=**T**)/**as.numeric**(**p98_2**))**,1**)**

mean_MFIs**<-**paste0**(**mean_MFIs_gc_train," [",mean_MFIs_gc_train2,

" x cutoff]"**)**

###########################################################################

# cutoff

cutoff2**<-**round**(**p98_2**)**

###########################################################################

# combine into 1 table

performance**<-**data.frame**(**cutoff2, spe_train, spe2_train, sen_train, sen2_train, sen2_train012,sen2_train34, mean_MFIs_gc_train,mean_MFIs_gc_train2, mean_MFIs, spe_test,spe2_test, sen_test, sen2_test,sen2_test012,sen2_test34, youden_test**)**

performance2**<-**performance**[**order**(**performance**$**sen_train,decreasing**=**T**)**,**]**

TableS3**<-**performance2**[**,c**(**1,3,5,6,7,10,17,12,14,15,16**)]**

colnames**(**TableS3**)[]<-**c**(**"Cutoff","Spe Training", "Sen Training",

"Sen Training Early", "Sen Training Late","Mean MFI","Youden Test",

"Spe Test", "Sen Test", "Sen Test Early", "Sen Test Late"**)**

View**(**TableS3**)**

Table2**<-**TableS3**[**1**:**13,c**(**"Spe Training", "Sen Training","Youden Test",

"Spe Test", "Sen Test"**)]**

View**(**Table2**)**

###########################################################################

###########################################################################

# Select best marker combinations in the training set

# select cases and controls training set

data_training**<-**data_binary**[**data_binary**$**trainingtest**==**"training",**]**

# select markers

markers**<-**colnames**(**data**[**5**:**68**])**

# two marker combinations -----------------------------------------------

marker_com**<-**combn**(**markers,2**)** # generate 2-marker combinations

cl **<-** makeCluster**(**4**)** # use four cores of the computer

clusterExport**(**cl,varlist**=**c**(**"marker_com","data_training"**))**

index**<-**parSapply**(**cl**=**cl,1**:**ncol**(**marker_com**)**,**function(**i**){**

dat**<-**data.frame**(**group**=**data_training**$**casecontrol,data_training**[**,marker_com**[**,i**]])**

col**<-**colnames**(**dat**)**

name**<-**paste0**(**col**[**2**]**,"+",col**[**3**])**

dat**$**combi**<-**dat**[**,2**]+**dat**[**,3**]**

dat**$**group2**<-**ifelse**(**dat**$**combi**==**0,0,1**)**

sen**<-**100*****sum**(**dat**$**group**==**1 **&** dat**$**group2**==**1**)/**155

spe**<-**100*****sum**(**dat**$**group**==**0 **&** dat**$**group2**==**0**)/**224

index**<-**sen**+**spe**-**100

out**<-**c**(**marker**=**name,youden**=**index,sensi**=**sen,speci**=**spe**)**

**})**

index2**<-**as.data.frame**(**t**(**index**))**

index2**[**,2**]<-**as.numeric**(**as.character**(**index2**[**,2**]))**

top2**<-**index2**[**order**(**index2**[**,2**]**,decreasing**=**T**)**,**]**

View**(**top2**)**

# three marker combinations ---------------------------------------------

marker_com**<-**combn**(**markers,3**)** # generate 3-marker combinations

cl **<-** makeCluster**(**4**)** # use four cores of the computer

clusterExport**(**cl,varlist**=**c**(**"marker_com","data_training"**))**

index**<-**parSapply**(**cl**=**cl,1**:**ncol**(**marker_com**)**,**function(**i**){**

dat**<-**data.frame**(**group**=**data_training**$**casecontrol,data_training**[**,marker_com**[**,i**]])**

col**<-**colnames**(**dat**)**

name**<-**paste0**(**col**[**2**]**,"+",col**[**3**]**,"+",col**[**4**])**

dat**$**combi**<-**dat**[**,2**]+**dat**[**,3**]+**dat**[**,4**]**

dat**$**group2**<-**ifelse**(**dat**$**combi**==**0,0,1**)**

sen**<-**100*****sum**(**dat**$**group**==**1 **&** dat**$**group2**==**1**)/**155

spe**<-**100*****sum**(**dat**$**group**==**0 **&** dat**$**group2**==**0**)/**224

index**<-**sen**+**spe**-**100

out**<-**c**(**marker**=**name,youden**=**index,sensi**=**sen,speci**=**spe**)**

**})**

index2**<-**as.data.frame**(**t**(**index**))**

index2**[**,2**]<-**as.numeric**(**as.character**(**index2**[**,2**]))**

top3**<-**index2**[**order**(**index2**[**,2**]**,decreasing**=**T**)**,**]**

View**(**top3**)**

# four marker combinations ----------------------------------------------

marker_com**<-**combn**(**markers,4**)** # generate 4-marker combination

cl **<-** makeCluster**(**4**)** # use four cores of the computer

clusterExport**(**cl,varlist**=**c**(**"marker_com","data_training"**))**

index**<-**parSapply**(**cl**=**cl,1**:**ncol**(**marker_com**)**,**function(**i**){**

dat**<-**data.frame**(**group**=**data_training**$**casecontrol,data_training**[**,marker_com**[**,i**]])**

col**<-**colnames**(**dat**)**

name**<-**paste0**(**col**[**2**]**,"+",col**[**3**]**,"+",col**[**4**]**,"+",col**[**5**])**

dat**$**combi**<-**dat**[**,2**]+**dat**[**,3**]+**dat**[**,4**]+**dat**[**,5**]**

dat**$**group2**<-**ifelse**(**dat**$**combi**==**0,0,1**)**

sen**<-**100*****sum**(**dat**$**group**==**1 **&** dat**$**group2**==**1**)/**155

spe**<-**100*****sum**(**dat**$**group**==**0 **&** dat**$**group2**==**0**)/**224

index**<-**sen**+**spe**-**100

out**<-**c**(**marker**=**name,youden**=**index,sensi**=**sen,speci**=**spe**)**

**})**

index2**<-**as.data.frame**(**t**(**index**))**

index2**[**,2**]<-**as.numeric**(**as.character**(**index2**[**,2**]))**

top4**<-**index2**[**order**(**index2**[**,2**]**,decreasing**=**T**)**,**]**

View**(**top4**)**

# five marker combinations ----------------------------------------------

marker_com**<-**combn**(**markers,5**)** # generate 5-marker combination

cl **<-** makeCluster**(**4**)** # use four cores of the computer

clusterExport**(**cl,varlist**=**c**(**"marker_com","data_training"**))**

index**<-**parSapply**(**cl**=**cl,1**:**ncol**(**marker_com**)**,**function(**i**){**

dat**<-**data.frame**(**group**=**data_training**$**casecontrol,data_training**[**,marker_com**[**,i**]])**

col**<-**colnames**(**dat**)**

name**<-**paste0**(**col**[**2**]**,"+",col**[**3**]**,"+",col**[**4**]**,"+",col**[**5**]**,"+",col**[**6**])**

dat**$**combi**<-**dat**[**,2**]+**dat**[**,3**]+**dat**[**,4**]+**dat**[**,5**]+**dat**[**,6**]**

dat**$**group2**<-**ifelse**(**dat**$**combi**==**0,0,1**)**

sen**<-**100*****sum**(**dat**$**group**==**1 **&** dat**$**group2**==**1**)/**155

spe**<-**100*****sum**(**dat**$**group**==**0 **&** dat**$**group2**==**0**)/**224

index**<-**sen**+**spe**-**100

out**<-**c**(**marker**=**name,youden**=**index,sensi**=**sen,speci**=**spe**)**

**})**

index2**<-**as.data.frame**(**t**(**index**))**

index2**[**,2**]<-**as.numeric**(**as.character**(**index2**[**,2**]))**

top5**<-**index2**[**order**(**index2**[**,2**]**,decreasing**=**T**)**,**]**

View**(**top5**)**

###########################################################################

# Calculate performance characteristics for the 11 best 5-marker

# combinations (Table 3)

# define 5-marker combinations

top11**<-**c**(**"TP53+SDCCAG8+ERBB2_C+MAGEA4+CTAG1", "TP53+ANXA4+ERBB2_C+MAGEA4+CTAG1", "TP53+SDCCAG8+MAGEA4+GRINA+CTAG1", "TP53+SDCCAG8+TPM3_iso3+MAGEA4+CTAG1", "TP53+DDX53+ERBB2_C+MAGEA4+CTAG1", "TP53+ERBB2_C+MAGEA4+CTAG1+PSCA", "TP53+ERBB2_C+MAGEA4+CTAG1+FOLH1_iso1", "TP53+ERBB2_C+MAGEA4+CTAG1+FOLH1_iso7", "TP53+ERBB2_C+MAGEA4+CTAG1+DCT", "TP53+SDCCAG8+DDX53+MAGEA4+CTAG1", "TP53+ERBB2_C+IGF2BP1+MAGEA4+CTAG1"**)**

data_binary**$**combi5a**<-**data_binary**$**TP53**+**data_binary**$**SDCCAG8**+**

data_binary**$**ERBB2_C**+**data_binary**$**MAGEA4**+**data_binary**$**CTAG1

data_binary**$**combi5b**<-**data_binary**$**TP53**+**data_binary**$**ANXA4**+**

data_binary**$**ERBB2_C**+**data_binary**$**MAGEA4**+**data_binary**$**CTAG1

data_binary**$**combi5c**<-**data_binary**$**TP53**+**data_binary**$**SDCCAG8**+**

data_binary**$**MAGEA4**+**data_binary**$**GRINA**+**data_binary**$**CTAG1

data_binary**$**combi5d**<-**data_binary**$**TP53**+**data_binary**$**SDCCAG8**+**

data_binary**$**TPM3_iso3**+**data_binary**$**MAGEA4**+**data_binary**$**CTAG1

data_binary**$**combi5e**<-**data_binary**$**TP53**+**data_binary**$**DDX53**+**

data_binary**$**ERBB2_C**+**data_binary**$**MAGEA4**+**data_binary**$**CTAG1

data_binary**$**combi5f**<-**data_binary**$**TP53**+**data_binary**$**ERBB2_C**+**

data_binary**$**MAGEA4**+**data_binary**$**CTAG1**+**data_binary**$**PSCA

data_binary**$**combi5g**<-**data_binary**$**TP53**+**data_binary**$**ERBB2_C**+**

data_binary**$**MAGEA4**+**data_binary**$**CTAG1**+**data_binary**$**FOLH1_iso1

data_binary**$**combi5h**<-**data_binary**$**TP53**+**data_binary**$**ERBB2_C**+**

data_binary**$**MAGEA4**+**data_binary**$**CTAG1**+**data_binary**$**FOLH1_iso7

data_binary**$**combi5i**<-**data_binary**$**TP53**+**data_binary**$**ERBB2_C**+**

data_binary**$**MAGEA4**+**data_binary**$**CTAG1**+**data_binary**$**DCT

data_binary**$**combi5j**<-**data_binary**$**TP53**+**data_binary**$**SDCCAG8**+**

data_binary**$**DDX53**+**data_binary**$**MAGEA4**+**data_binary**$**CTAG1

data_binary**$**combi5k**<-**data_binary**$**TP53**+**data_binary**$**ERBB2_C**+**

data_binary**$**IGF2BP1**+**data_binary**$**MAGEA4**+**data_binary**$**CTAG1

data_binary**$**combi5a2**<-**ifelse**(**data_binary**$**combi5a**==**0,0,1**)**

data_binary**$**combi5b2**<-**ifelse**(**data_binary**$**combi5b**==**0,0,1**)**

data_binary**$**combi5c2**<-**ifelse**(**data_binary**$**combi5c**==**0,0,1**)**

data_binary**$**combi5d2**<-**ifelse**(**data_binary**$**combi5d**==**0,0,1**)**

data_binary**$**combi5e2**<-**ifelse**(**data_binary**$**combi5e**==**0,0,1**)**

data_binary**$**combi5f2**<-**ifelse**(**data_binary**$**combi5f**==**0,0,1**)**

data_binary**$**combi5g2**<-**ifelse**(**data_binary**$**combi5g**==**0,0,1**)**

data_binary**$**combi5h2**<-**ifelse**(**data_binary**$**combi5h**==**0,0,1**)**

data_binary**$**combi5i2**<-**ifelse**(**data_binary**$**combi5i**==**0,0,1**)**

data_binary**$**combi5j2**<-**ifelse**(**data_binary**$**combi5j**==**0,0,1**)**

data_binary**$**combi5k2**<-**ifelse**(**data_binary**$**combi5k**==**0,0,1**)**

# Calculate number of positive and negative test results for each group

a5**<-**table**(**data_binary**$**combi5a2,data_binary**$**group**)**

b5**<-**table**(**data_binary**$**combi5b2,data_binary**$**group**)**

c5**<-**table**(**data_binary**$**combi5c2,data_binary**$**group**)**

d5**<-**table**(**data_binary**$**combi5d2,data_binary**$**group**)**

e5**<-**table**(**data_binary**$**combi5e2,data_binary**$**group**)**

f5**<-**table**(**data_binary**$**combi5f2,data_binary**$**group**)**

g5**<-**table**(**data_binary**$**combi5g2,data_binary**$**group**)**

h5**<-**table**(**data_binary**$**combi5h2,data_binary**$**group**)**

i5**<-**table**(**data_binary**$**combi5i2,data_binary**$**group**)**

j5**<-**table**(**data_binary**$**combi5j2,data_binary**$**group**)**

k5**<-**table**(**data_binary**$**combi5k2,data_binary**$**group**)**

perf5a**<-**rbind**(**a5,b5,c5,d5,e5,f5,g5,h5,i5,j5,k5**)**

n_pos**<-**perf5a**[**c**(**2,4,6,8,10,12,14,16,18,20,22**)**,**]**

n_neg**<-**perf5a**[**c**(**1,3,5,7,9,11,13,15,17,19,21**)**,**]**

# calculate sensitivity, specificity and Youden's index

n_total**<-**table**(**data_binary**$**group**)**

n_total2**<-**matrix**(**rep**(**n_total,11**)**,nrow**=**11, byrow**=**T**)**

sen**<-**round**(**100***(**n_pos**/**n_total2**))**

spe**<-**round**(**100***(**n_neg**/**n_total2**))**

youden_training**<-**round**(((**n_pos**/**n_total2**)[**,"GC_dp"**])+**

**((**n_neg**/**n_total2**)[**,"controls_blitz"**])-**1,2**)**

youden_test**<-**round**(((**n_pos**/**n_total2**)[**,"GC_esther2verdi"**])+**

**((**n_neg**/**n_total2**)[**,"controls_e1_bl"**])-**1,2**)**

# calculate confidence intervals for sensitivity and specificity

cis_sensi_lower**<-**matrix**(**round**((**binconf**(**n_pos,n_total2**)***100**))[**,2**]**,nrow**=**11**)**

cis_sensi_upper**<-**matrix**(**round**((**binconf**(**n_pos,n_total2**)***100**))[**,3**]**,nrow**=**11**)**

sen2**<-**matrix**(**paste0**(**sen," (",cis_sensi_lower,"-",cis_sensi_upper,

")"**)**,nrow**=**11**)**

colnames**(**sen2**)<-**colnames**(**sen**)**

cis_speci_lower**<-**matrix**(**round**((**binconf**(**n_neg,n_total2**)***100**))[**,2**]**,nrow**=**11**)**

cis_speci_upper**<-**matrix**(**round**((**binconf**(**n_neg,n_total2**)***100**))[**,3**]**,nrow**=**11**)**

spe2**<-**matrix**(**paste0**(**spe," (",cis_speci_lower,"-",cis_speci_upper,

")"**)**,nrow**=**11**)**

colnames**(**spe2**)<-**colnames**(**spe**)**

# combine into 1 table

Table3**<-**cbind**(**top11,youden_training,sen2**[**,"GC_dp"**]**,spe2**[**,"controls_blitz"**]**,

youden_test,sen2**[**,"GC_esther2verdi"**]**,spe2**[**,"controls_e1_bl"**])**

colnames**(**Table3**)<-**c**(**"marker","youden_train", "sen_train", "spe_train","youden_test","sen_test" ,"spe_test"**)**

View**(**Table3**)**

###########################################################################
